# A randomized controlled trial of adjunctive speleotherapy in asthma, COPD and long COVID

**DOI:** 10.1038/s41598-026-52301-4

**Published:** 2026-05-22

**Authors:** Joachim Schwarz, Madelaine Eicke, Nina Schwedler, Gerrit von Komorowski, Verena Goldfuss, Wolfgang Fladerer, Béatrice Barbolan, Martin Mogk, Natascha Sommer

**Affiliations:** 1 German Speleotherapy Association, Marktplatz 3, 75387 Neubulach, Germany; 2 Justus Liebig University Giessen, Ludwigstrasse 23, 35390 Giessen, Germany; 3 Tiefer Stollen, Erzhäusle 1, 73433 Aalen, Germany; 4 Unità Operativa Di Anestesia E Rianimazione, Ospedale di Saronno Piazza Borella, 1, 21047 Saronno, VA Italy; 5 moreDATA GmbH, Neuen Bäue 22, 35390 Giessen, Germany; 6 Department of Internal Medicine, Justus Liebig University Giessen, Medical Clinic and Polyclinic II, Klinikstrasse 33, 35392 Giessen, Germany; 7 Excellence Cluster Cardio-Pulmonary Institute (CPI), Universities of Giessen and Marburg Lung Center (UGMLC), Member of the German Center for Lung Research (DZL), Institute of Lung Health (ILH), Munich, Germany

**Keywords:** Speleotherapy, Asthma, COPD, Long-COVID, Dysfunctional breathing, Diseases, Health care, Medical research, Signs and symptoms

## Abstract

Speleotherapy (underground climate therapy) is a non-pharmacological intervention for chronic respiratory diseases. This randomized controlled trial investigated whether a 3-week speleotherapy course (6 sessions, 2 h/week) improves respiratory outcomes in patients on standard background therapy with asthma, COPD, or Long COVID, and whether it affects blood CO_2_ levels. The control group did not receive speleotherapy but continued their standard therapy. A total of 208 patients (asthma: n = 107; COPD: n = 59; Long COVID: n = 42) were enrolled across nine centers in Germany, Austria, and Italy. Assessments were conducted pre-intervention (T1), post-intervention (T2), and at 3-month follow-up (T3). Outcome measures included airway inflammation (FeNO), pulmonary function parameters (FVC% predicted values, FEV₁% predicted values, FEV₁/FVC, PEF% predicted values), and respiratory muscle strength (MIP and MEP in absolute values). In addition the following validated questionnaires were administered: Asthma Control Test (ACT), Asthma Quality of Life Questionnaire (AQLQ), COPD Assessment Test (CAT), St. George’s Respiratory Questionnaire (SGRQ), Nijmegen Questionnaire (NQ), and Fatigue Assessment Scale (FAS), along with the Long COVID questionnaire from the Median Clinic Group. CO_2_ levels were assessed via capillary blood (SpCO_2_) and end-tidal CO_2_ (PetCO_2_). Between-group comparisons used the Mann–Whitney U test; within-group changes were assessed with the Wilcoxon signed-rank test (Bonferroni-Holm corrected). In patients with asthma, the predefined primary endpoint (FeNO) showed no significant improvement. In contrast, patient-reported outcomes improved significantly, with clinically relevant gains in asthma control (ACT: p < 0.001) and asthma-related quality of life (total AQLQ: p = 0.005). Lung function parameters showed statistically significant but modest improvements at T2 (FVC: p = 0.011; PEF: p = 0.010; FEV₁ in participants < 70 years: p = 0.035). In patients with COPD, symptom burden improved according to CAT scores (p = 0.036), while no improvements in lung function were observed. Patients with Long COVID reported significant improvements in dysfunctional breathing (NQ: T2: p = 0.014), dyspnea (T2: p = 0.026; T3: p = 0.001), and “problems with stair climbing/muscle exertion” (T2: p = 0.042), as well as improvements in anxiety and sleep-related symptoms (T2: p = 0.021). No improvements in lung function were observed in this group. In the total cohort, the intervention group showed statistically significant improvements compared to controls in respiratory muscle strength (MIP: p = 0.002; MEP: p = 0.018) and dysfunctional breathing (NQ scores at T2: p = 0.007; T3: p = 0.017). In CO₂-rich speleotherapy centers, both SpCO₂ (p = 0.026) and PetCO₂ (p < 0.001) increased at T2. While speleotherapy did not improve FeNO, it was associated with clinically relevant improvements in patient-reported outcomes across disease groups. Changes in lung function and respiratory muscle strength were statistically significant but modest and should be interpreted with caution. Overall, speleotherapy may have direct effects on the airways and breathing regulation, with more consistent evidence for improvements in breathing patterns than for direct effects on the airways.

**Trial registration**: DRKS00033365 (retrospectively registered).

**Supplementary Information:**

The online version contains supplementary material available at 10.1038/s41598-026-52301-4.

## Introduction

Chronic respiratory diseases are among the leading causes of morbidity and mortality worldwide^1^. In 2023, the prevalence of chronic obstructive pulmonary disease (COPD) in Germany was 6,73%^2^, and the prevalence of bronchial asthma was 4.35%^3^. For the years 2020 and 2021, the global prevalence of Long COVID was estimated at 6.2%, with respiratory involvement reported in 3.7% of cases^4^.

While pharmacological treatment options for COPD remain limited and the pathophysiology and diagnostics of Long COVID are incompletely understood, the quality of life for patients with asthma can be significantly improved through pharmacological treatment, environmental factors, and general medical care. Nevertheless, the disease burden remains substantial due to the high prevalence^5^.

Non-pharmacological therapeutic approaches constitute an important complementary pillar. These include patient education, physical training, respiratory physiotherapy, smoking cessation, weight control, avoidance of air pollutants, and psychosocial interventions^6^.

Another approach is speleotherapy, a climate-based treatment conducted in natural caves or decommissioned mines. Patients typically spend 2 h per day lying in a sleeping bag, 6 days per week, over 3 weeks in an underground therapy area^7^. Certified speleotherapeutic facilities must meet strict climatic criteria (Table 1) to be recognized as location-specific therapeutic remedies as part of official health resort accreditation in Germany^8^.

**Table 1:** Quality criteria for the microclimate in a certified cold-air speleotherapy area (Heilstollen) in Germany.

Quality parameter	Value
Relative humidity	> 85%
Temperature	5–12 °C
Particulate Matter (PM10)	< 10 µg/m^3^
Nitrogen Dioxide (NO₂)	< 10 µg/m^3^
Sound Level	< 30 dB

This form of therapy has a long tradition in Central and Eastern Europe as well as Western Asia, where it is used as a complementary treatment for allergic respiratory diseases^9,10^.

The following mechanisms of action have been proposed, although clear scientific evidence is lacking:

- Reduction of inhaled irritants due to extremely pure air free from pollen, dust, and spores
- Thermophysical effect: Cold, humid air is warmed within the airways, potentially drawing fluid from the mucosa and reducing oedema.
- The increased CO_2_ concentration in the air (0.08% to 0.4%, compared to 0.04% in ambient air) may promote relaxation of the respiratory muscles.

The evidence supporting the efficacy of speleotherapy in asthma is limited to uncontrolled interventional studies and one randomized controlled trial (RCT) involving children aged 4 to 10^11^. This study demonstrated a significant improvement in FEV_1_ of 10.5 percentage points in the intervention group versus 0.0 percentage points in the control group (p = 0.0002). A significant effect was also observed in 12 out of 25 secondary outcome measures. However, no randomized studies involving adults have been conducted so far, and evidence for the long-term efficacy of speleotherapy is lacking.

The present study aimed to evaluate the effects of speleotherapy in adult patients with asthma, COPD, and Long COVID in a multicentre RCT—immediately after the intervention and 3 months later. Additionally, the influence of CO_2_ levels in the therapeutic cave environment on ventilatory parameters (petCO2, pCO2) was analyzed. In addition, the study monitored adverse events and unintended effects during the intervention period.

This study addressed two central research questions:

- Primary question: Does a 3-week speleotherapy program (six sessions per week, each lasting 2 h) lead to an improvement in disease status in patients with chronic respiratory diseases?
- Secondary question: Does speleotherapy lead to an increase in blood pCO2 levels and end-tidal CO2 (PetCO2) in exhaled air?

The primary research question was examined across three diagnostic groups: bronchial asthma, COPD, and Long COVID. The secondary question was assessed as part of a parallel subproject.

## Methods

### Study design and setting

This study was designed as a multicentre, parallel-group, randomized controlled superiority trial with a 1:1 allocation ratio.

Nine speleotherapy facilities in Germany, Austria, and South Tyrol (Italy) were included to address the primary research question. Five of these sites (marked with *) also participated in the CO2 subproject:

- Grube Bindweide, Rhineland-Palatinate *
- Silberbergwerk Bodenmais, Bavaria *
- Tiefer Stollen Aalen, Baden-Württemberg
- Silberbergwerk Neubulach, Baden-Württemberg *
- Eisensteinstollen Bad Grund, Lower Saxony
- Kluterthöhle Ennepetal, North Rhine-Westphalia *
- Feengrotten Saalfeld, Thuringia
- Klimastollen Prettau, South Tyrol, Italy *
- Friedrichstollen Bad Bleiberg, Austria

All facilities met the required climatic criteria and followed a standardized treatment protocol based on prior empirical experience.

Patients lay on comfortable reclining chairs for 1.5–2 h, wrapped in sleeping bags. At the beginning of each session, general physical and breathing exercises were performed for 5–10 min (7).

Data collection occurred at three time points: pre-treatment (T1), immediately post-treatment (T2), and 3 months after treatment completion (T3), or at corresponding time points for the control group.

The random allocation sequence was generated centrally using a computer-based random number generator.

No changes to the trial protocol, outcomes, or prespecified analyses were made after trial commencement. No interim analyses were planned or performed, and no formal stopping guidelines were defined.

Patients and the public were not involved in the design, conduct, reporting, or dissemination plans of this trial.

### Eligibility criteria and patient information

Participants aged 18 to 80 years with a physician-confirmed diagnosis of bronchial asthma, chronic obstructive pulmonary disease (COPD), or Long COVID were eligible for inclusion in this study. Recruitment was conducted through outpatient settings, and interested individuals with one of these diagnoses were able to register for participation.

The primary basis for study inclusion was the written diagnosis provided by the participant’s general practitioner or treating physician. No additional diagnostic verification by study physicians or specialists was performed as part of the study protocol.

Spirometry was performed primarily for follow-up monitoring and was not used as an inclusion criterion. Measurements were obtained under routine outpatient conditions and were conducted without standardized bronchodilator administration. Medication regimens and timing of intake were not controlled. Consequently, some participants classified as having COPD showed lung function values above commonly applied thresholds (e.g., FEV₁/FVC > 0.7). This may be attributable to current treatment effects, diagnostic variability in routine outpatient care, or intra-individual variability in lung function parameters (Additional File 5). Diagnostic group assignment therefore relied on documented physician diagnoses rather than spirometric cut-off values.

Exclusion criteria included the following: COPD (Global Initiative for Chronic Obstructive Lung Disease [GOLD]) stage 4, uncontrolled asthma (Global Initiative for Asthma [GINA] grade 3), frequent exacerbations (≥ 2 exacerbations requiring change in medication), decompensated heart failure, claustrophobia, pregnancy, acute upper respiratory infections (< 4 weeks prior to inclusion), active smoking within the past 3 months, and participation in speleotherapy within the previous year.

Following comprehensive written patient information and assessment of eligibility via a questionnaire, participants provided written informed consent for participation and data processing. Treatment was conducted on an outpatient basis; participants of the interventional group travelled daily from their place of residence (or temporary accommodation) to the therapy site, covering an average distance of 15 km. The control group received no speleotherapy during the study period but was allowed to participate after the study concluded.

### Sample size estimation

The sample size was calculated based on the change between two measurement points for the primary endpoints and the two arms, assuming a medium to large effect (Cohen’s d ≈ 0.8), with α = 0.05 (two-sided) and a power of 90%. A total of n = 42 patients per arm (intervention/control) for each subgroup (Asthma, COPD, Long-COVID) was determined to account for non-parametric analysis methods and expected dropouts (15% and 10%, respectively).

### Randomization

Within each centre for each disease randomization was stratified by sex and treatment without additional blocking. Allocation concealment was ensured by central randomisation; investigators responsible for participant enrolment did not have access to the random allocation sequence and were informed of group assignment only after enrolment had been completed. It was conducted up to 2 weeks before the start of the study. In later phases, all eligible patients were included to meet the target sample size, which may have led to imbalances in sex and diagnosis distributions. Due to the nature of the intervention, participants and care providers were not blinded to group allocation. Outcome assessors and data analysts were not blinded.

### Outcome measures and instruments

#### Primary endpoints

(Lung function parameters (FEV₁, FVC, and PEF) are expressed as percent predicted values, whereas MIP and MEP are reported as absolute pressure values.)

- Asthma: Change in type 2 inflammation measured via fractional exhaled nitric oxide (FeNO, in ppb; device: NIOX VERO von NIox)
- COPD: Improvement in lung function (forced vital capacity [FVC], forced expiratory volume in 1 s [FEV₁], ratio of FEV₁ to FVC [FEV₁/FVC], peak expiratory flow [PEF]) and respiratory muscle strength (maximal inspiratory pressure [MIP] and maximal expiratory pressure [MEP], measured with the Pneumotrac RMS).
- Long COVID: Improvement in lung function (FEV₁, FVC, PEF) and respiratory muscle strength (MIP and MEP).
- CO_2_ Subproject: Increase in end-tidal CO_2_ (PetCO_2_) (device: Masimo RAD-97)

#### Secondary endpoints

- Asthma: Lung function (as above); questionnaires: Asthma Control Test (ACT), Asthma Quality of Life Questionnaire (AQLQ), Nijmegen Questionnaire (NQ)
- COPD: Questionnaires: COPD Assessment Test (CAT), St. George’s Respiratory Questionnaire (SGRQ)
- Long COVID: Questionnaires: Patient questionnaire on Long COVID syndrome (Median Clinic Group), Fatigue Assessment Scale (FAS), and NQ (for dysfunctional breathing)
- CO_2_ Subproject: Partial Pressure of Carbon Dioxide (pCO_2_) in capillary blood (measured via EPOC Blood Analysis System, Siemens)

In the **CO**_2_ **subproject** in all five participating speleotherapy centres, the PetCO_2_, respiratory rate, and SpCO_2_ were measured on one therapy day before, during, and directly after the treatment session.

### Questionnaires used

- ACT: 5 items. Assesses how well asthma is controlled^12^.
- AQLQ: 32 items on a 7-point scale. Measures the extent to which asthma affects emotional and physical well-being^13^.
- CAT: 8 items on a 6-point scale. Evaluates the severity and impact of COPD on the patient’s daily life^14^.
- SGRQ: 50 items. Assesses disease-related impairments in patients with chronic respiratory diseases^15^.
- NQ: 16 items; cut-off score > 20. Assesses the degree of dysfunctional breathing^16^.
- FAS: 10 items. Used to assess the presence and severity of chronic fatigue^17^.
- Patient Questionnaire on Long COVID Syndrome (developed by the Median Clinic Group): 14 symptom-specific items with four response categories to rate severity. The Long-COVID Questionnaire was used with written permission from MEDIAN Klinik Flechtingen (Dr. P. O. Schüller, 2023). As this instrument is not formally published, it is cited as an unpublished clinical assessment tool^18^.

Additional file 1 provides an overview table of all measurement parameters, indicating whether they were defined as primary or secondary endpoints. It also summarizes the instruments used, the corresponding measurement scales, how the assessments were performed, and by whom the measurements were conducted.

### Harms

Harms were defined as any adverse events or unintended symptoms occurring during the intervention period. Adverse events were assessed non-systematically by patient self-report during therapy sessions and at follow-up visits.

### Ethics

All methods were performed in accordance with the relevant guidelines and regulations and complied with applicable ethical and legal standards. The study was approved (or classified as not requiring formal review) by the ethics committees of the State Chambers of Physicians in Rhineland-Palatinate (initial vote), Baden-Württemberg, Bavaria, Thuringia, Westphalia-Lippe, and Lower Saxony, as well as by the responsible ethics committees in Italy (South Tyrol) and Austria (Carinthia). Registration: retrospectively registered on July 24, 2025). The study was conducted in accordance with the General Data Protection Regulation (GDPR, EU Regulation 2016/679).

### Data protection

All participants received written information regarding the study’s objectives, procedures, and data processing. Data collection was conducted using pseudonymized identifiers. The randomization list was stored separately with password protection.

### Statistical methods

Metric and ordinal data were presented descriptively in tables, including mean, standard deviation, and 95% confidence intervals for the mean. As alternative measures of central tendency and dispersion, the median (50th percentile) and interquartile range (IQR) were reported. Missing data were assessed across all primary outcomes and time points. As the overall rate of missingness was low (below 10%), no imputation method was applied. All analyses were therefore based on complete case analysis. Participants were analysed in the groups to which they were originally randomised. All continuous outcomes were summarised as medians with interquartile ranges and analysed as changes from baseline. Outcomes were assessed at baseline (T1), immediately after the intervention (T2), and at 3-month follow-up (T3).

Normal distribution was assessed using Q-Q plots and the Shapiro–Wilk test. The results typically contradicted the assumption of normality; therefore, data were analysed using the nonparametric approach for longitudinal data by Brunner and Langer^19^ to evaluate the main effects of treatment and time, as well as their interaction. Significant effects were further examined using post-hoc comparisons: comparisons between two groups and the change between two measurement points were conducted using the non-parametric Mann–Whitney U test and Wilcoxon signed-rank tests for within-group changes over time. The results of the repeated measures analysis according to Brunner and Langer, as well as a scheme of the post-hoc analysis for group and time effects, are presented in the additional file 9.

To control for type I error inflation, p-values were adjusted using the Bonferroni-Holm procedure. Given evidence suggesting a reduced therapeutic response in older patients with asthma^20^, a post hoc subgroup analysis was conducted in asthma patients aged under 70 years using the same statistical methods as in the primary analysis. This analysis was exploratory and not prespecified.

The results were graphically visualized using boxplots. Data analysis was performed using R for Windows, version 4.4.1.

## Results

### Study population

Of the 233 individuals initially enrolled, 208 patients with a diagnosis of bronchial asthma, COPD, or Long COVID were included in the analysis. Participants were recruited between 25 May 2024 and 15 September 2024, and follow-up for all outcomes was completed on 10 January 2025. A total of 25 participants were excluded for the following reasons: illness prior to the start of therapy (n = 6), illness during therapy (n = 5), fewer than 12 therapy sessions completed (n = 7), ineligible diagnosis (n = 6), and other reasons (n = 1).

Exclusions due to ineligible diagnoses were necessary based on subsequent diagnostic information. These included fibrosis (n = 1), allergic rhinitis (n = 1), multiple allergies (n = 1), post-infectious bronchitis (n = 1), a positive COVID-19 test without symptoms suggestive of Long COVID (n = 1), and a medically unconfirmed suspected COVID-19 case without Long COVID symptoms (n = 1).The average age of the 208 included patients was 62 (females: 59.2; males: 65.6). Additional file 2 shows the distribution of participants by diagnosis, sex, and group allocation (intervention/control).

The intervention was delivered by trained medical staff at the participating speleotherapy centres. Patient adherence was high, as reflected by a low rate of missing data of approximately 10%. Concomitant care, including regular pharmacological treatment, was continued unchanged in both the intervention and control groups throughout the study period.

### Total cohort: Respiratory muscle strength (MIP, MEP) is improved after speleotherapy

In the total cohort, patients of the speleopherapy group (n = 98) and control group (n = 110) did not differ in baseline medication, FeNO, lung functional parameters, respiratory muscle strength (MIP, MEP) or questionnaire based scores at T1 before start of speleotherapy (Additional file 3 and 4). After 3 weeks of therapy (T2-T1) lung function parameters FVC and FEV_1_improved significantly in both groups, whereas PEF only improved significantly in the interventional group. Moreover, respiratory muscle strength improved only in the interventional group and was significantly higher at T2 compared to the control group. Dysfunctional breathing according to the Nijmegen Questionnaire (NQ) decreased significantly in the interventional group at T2 and T3 and also in comparison to the control group. FeNO was neither changed in control, nor in the interventional group (Additional file 3).

### Asthma: FeNO did not improve, whereas lung function, respiratory muscle strength, and patient-reported outcomes did after speleotherapy

In the total cohort of patients with bronchial asthma (n = 107; intervention: 54; control: 53), there were no differences between the intervention and control groups at baseline with regard to medication, FeNO, lung function, or respiratory muscle strength (MIP, MEP) (Additional file 4 and 5).

#### Primary endpoints

FeNO, the pre-defined primary endpoint, did not improve but remained stable in both groups throughout the study (Additional file 5).

#### Secondary endpoints

**Fig. 1:**
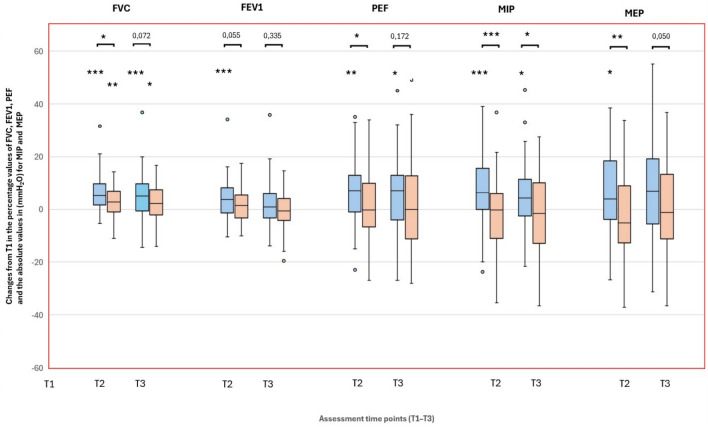
Pulmonary function and respiratory muscle strength (MIP, MEP) after speleotherapy in asthma (blue: intervention, orange: control). Changes in pulmonary function and respiratory muscle strength in patients with asthma after speleotherapy (I – blue boxes) and without speleotherapy (C – orange boxes). Parameters: FVC (forced vital capacity) in % predicted values, FEV_1_ (forced expiratory volume in 1 s) in % predicted values, PEF (peak expiratory flow) in % predicted values, MIP (maximal inspiratory pressure) absolute values, MEP (maximal expiratory pressure) absolute values. Assessments were performed at three time points: T1 – before speleotherapy; T2 – immediately after three weeks with (I) or without (C) speleotherapy; T3 – three months after (I) or without (C) speleotherapy. Sample size (N): T1 – 54 (I), 53 (C); T2 – 52 (I), 52 (C); T3 – 51 (I), 50 (C). Wilcoxon test for within-group comparisons across time (*p < 0.05, **p < 0.01, ***p < 0.001); Mann–Whitney U test for between-group comparisons (*p < 0.05, **p < 0.01, ***p < 0.001); extreme values are not displayed for clarity in PEF T2 C-group, MIP T3 C-group, MEP T2 both groups, and MEP T3 C-group.

After 3 weeks (T2-T1), lung function parameters improved significantly in the intervention group across almost all parameters (Fig. 1, Additional file 5). Importantly, FVC and PEF showed significant improvements in the total group, and FEV_1_ in patients younger than 70 years compared to the control group (Fig. 1, Additional file 5). In both groups, the sustained increase in FVC at T3 was accompanied by a reduction in the Tiffeneau Index (FEV_1_/FVC) at T3 (Additional file 5).

Respiratory muscle strength (MIP, MEP) improved in the intervention group at T2, with MIP remaining improved at T3 (Fig. 1, Additional file 5). These changes were significant compared to the control group (Additional file 5).

**Fig. 2:**
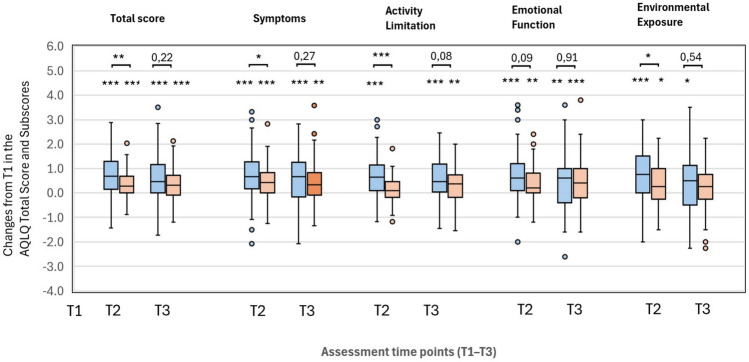
Changes in AQLQ scores after speleotherapy in asthma (blue: intervention; orange: control). Changes in Asthma Quality of Life Questionnaire (AQLQ) in patients with asthma after speleotherapy (I – blue boxes) and without speleotherapy (C – orange boxes). Parameters: total score, symptoms, activity limitation, emotional function, and environmental exposure. Assessments were performed at three time points: T1 – before speleotherapy; T2 – immediately after three weeks with (I) or without (C) speleotherapy; T3 – three months after (I) or without (C) speleotherapy. Sample sizes (N): T1 – 54 (I), 53 (C); T2 – 54 (I), 51 (C); T3 – 53 (I), 51 (C). Wilcoxon test for within-group comparisons across time (*p < 0.05, **p < 0.01, ***p < 0.001); Mann–Whitney U test for between-group comparisons (*p < 0.05, **p < 0.01, ***p < 0.001). Extreme values are not displayed for clarity in “environmental exposure” at T2 and “activity limitation” at T3 in the I group.

Regarding the questionnaires for dysfunctional breathing (NQ), asthma control (ACT), and quality of life (AQLQ), the intervention group demonstrated significant improvements at both T2 and T3 (Additional file 5, Figs. 2 and 3). Between-group comparisons showed superior ACT and AQLQ total scores in the intervention group at T2 (Additional file 5). In particular, symptoms, activity limitation, and environmental exposure was improved compared to controls (Fig. 2). In participants under 70 years of age, dysfunctional breathing measured by the Nijmegen Questionnaire (NQ) showed a significant decrease in the intervention group at T2 and T3, as well as compared with the control group (Additional file 5).

**Fig. 3:**
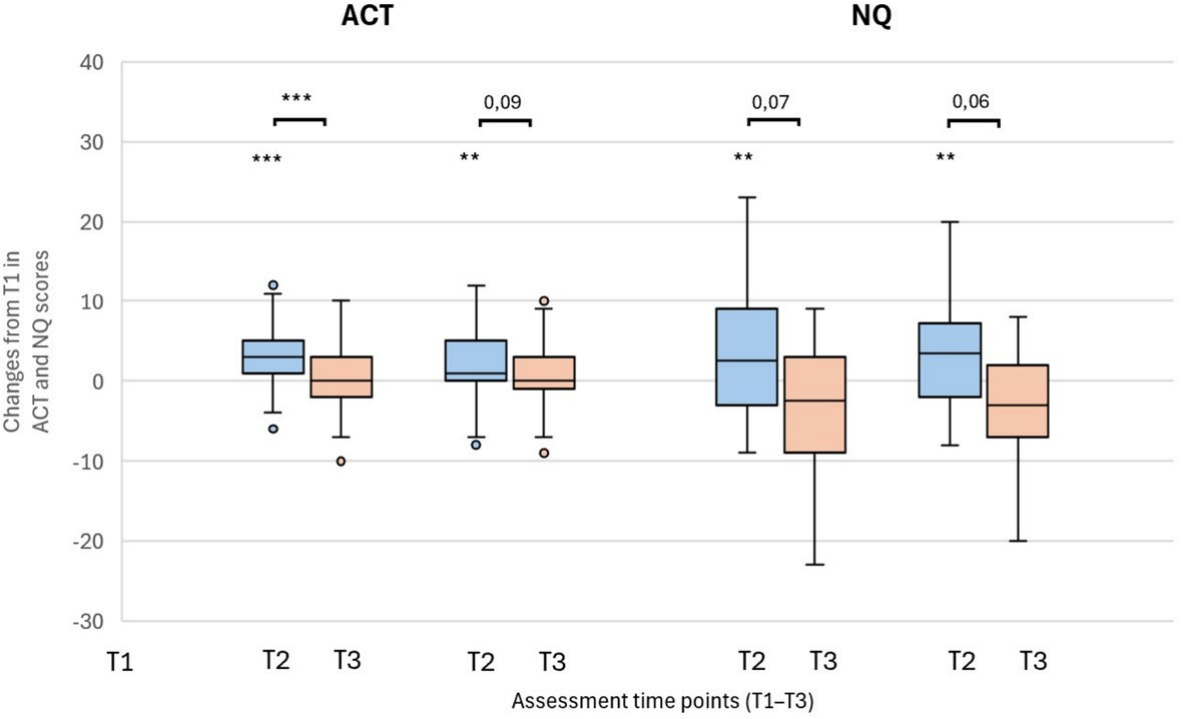
Changes in ACT and NQ scores after asthma (blue: intervention; orange: control). Changes in the total score of Asthma Control Test (ACT) and in Nijmegen Questionaire (NQ) in patients with asthma after speleotherapy (I – blue boxes) and without speleotherapy (C – orange boxes). Assessments were performed at three time points: T1 – before speleotherapy; T2 – immediately after three weeks with (I) or without (C) speleotherapy; T3 – three months after speleotherapy (I) or without speleotherapy (C). Sample sizes (N): T1 – 54 (I), 53 (C); T2 – 54 (I), 52 (C); T3 – 54 (I), 51 (C) in ACT and 52 (C) in NQ. Wilcoxon test for within-group comparisons across time (*p < 0.05, **p < 0.01, ***p < 0.001); Mann–Whitney U test for between-group comparisons (*p < 0.05, **p < 0.01, ***p < 0.001).

### COPD: No improvement in lung function was observed, but the CAT questionnaire score improved

In the total cohort (n = 59; intervention: 27; control: 32), patients in the intervention and control groups did not differ in baseline medication, vital signs, FeNO, FVC, PEF or respiratory muscle strength (MIP, MEP). FEV_1_at baseline was significantly lower in the intervention group compared to the control group (Additional file 4 and 6).

#### Primary endpoints

After 3 weeks of therapy (T2-T1), FVC improved in the control group, while the FEV_1_/FVC ratio declined in both groups. There were no differences in lung function and respiratory muscle strength (MIP, MEP) between the groups.

#### Secondary endpoints

**Fig. 4:**
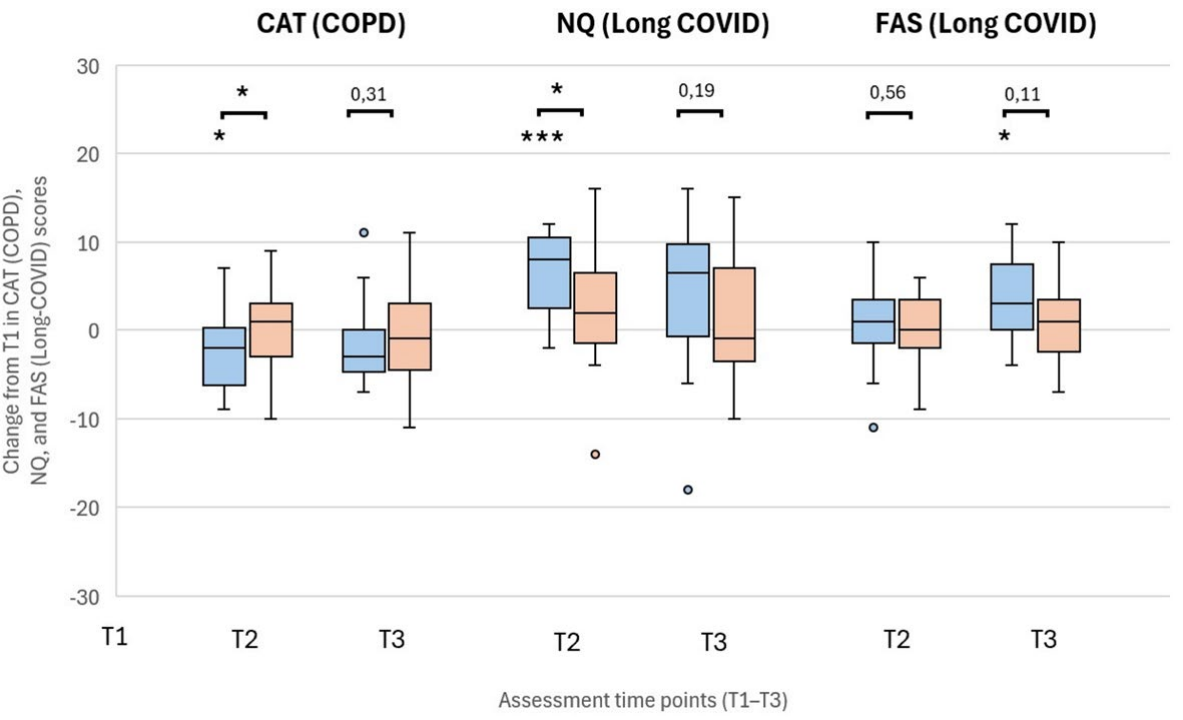
Changes in CAT (COPD), NQ and FAS (Long COVID) after speleotherapy (blue: intervention; orange: control group). Changes in COPD Assessment Test (CAT) in patients with COPD and Nijmegen questionnaire (NQ) and Fatigue Assessment Scale (FAS) in patients with Long-COVID after speleotherapy (I – blue boxes) and without speleotherapy (C – orange boxes). Assessments were performed at three time points: T1 – before speleotherapy; T2 – immediately after three weeks with (I) or without (C) speleotherapy; T3 – three months after speleotherapy (I) or without speleotherapy (C). Sample sizes CODP (N): T1 – 26 (I), 31 (C); T2 – 26 (I), 31 (C); T3 – 24 (I), 28 (C). Sample sizes Long-COVID (NQ): T1 – 17 (I), 25 (C); T2 – 17 (I), 25 (C); T3 – 16 (I), 24 (C). Sample sizes Long-COVID (FAS): T1 – 17 (I), 23 (C); T2 – 17 (I), 24 (C); T3 – 16 (I), 23 (C). Wilcoxon test for within-group comparisons across time (*p < 0.05, **p < 0.01, ***p < 0.001); Mann–Whitney U test for between-group comparisons (*p < 0.05, **p < 0.01, ***p < 0.001). Extreme values are not displayed for clarity in NQ at T2 in the I group.

The COPD Assessment Test (CAT) showed significant improvements in the intervention group at T2 and also in comparison to the control group (Fig. 4). The St. George’s Respiratory Questionnaire (SGRQ) showed no differences between the two groups (Additional file 6).

### Long COVID: No lung-function improvement, but dysfunctional breathing improved after speleotherapy

In the total cohort of patients with Long COVID (n = 42; intervention: 17; control: 25), there were no differences in baseline medication, vital signs, FeNO, lung functional parameters, respiratory muscle strength (MIP, MEP) or questionnaire-based scores at T1 (Additional file 4 and 7).

#### Primary endpoints

After 3 weeks of therapy (T2), lung function parameters (FEV_1_, PEF) improved within the control group, with FEV_1_ also in comparison to the intervention group.

#### Secondary endpoints

NQ scores, indicative of dysfunctional breathing, decreased in the intervention group at T2 compared to the control group, and a reduction in fatigue (FAS) was observed in the intervention group at T3 (Fig. 4). According to the Long COVID Questionnaire, the intervention group reported significant improvements over the control group in three of fourteen domains—dyspnoea, problems with stair climbing/ muscle exertion, and anxiety/sleep disturbances—at T2, with the improvement in dyspnoea persisting at T3 (Additional file 7).

### ***An increased CO***_***2***_*** concentration in the therapy cave leads to an increased CO***_***2***_*** concentration in the exhaled air***

During the therapy period, CO_2_ concentrations of the caves were measured in a sampling basis. At Bodenmais, only a slight increase was observed (462 ppm) compared to outside air (400 ppm). Other values recorded were: Prettau (970 ppm), Neubulach (1705 ppm), Ennepetal (2500 ppm), Bindweide (3665 ppm).

To assess the significance of the increased CO_2_ concentration in the therapy cave for ventilation, the following investigations were carried out in these five therapeutic caves.

**Fig. 5:**
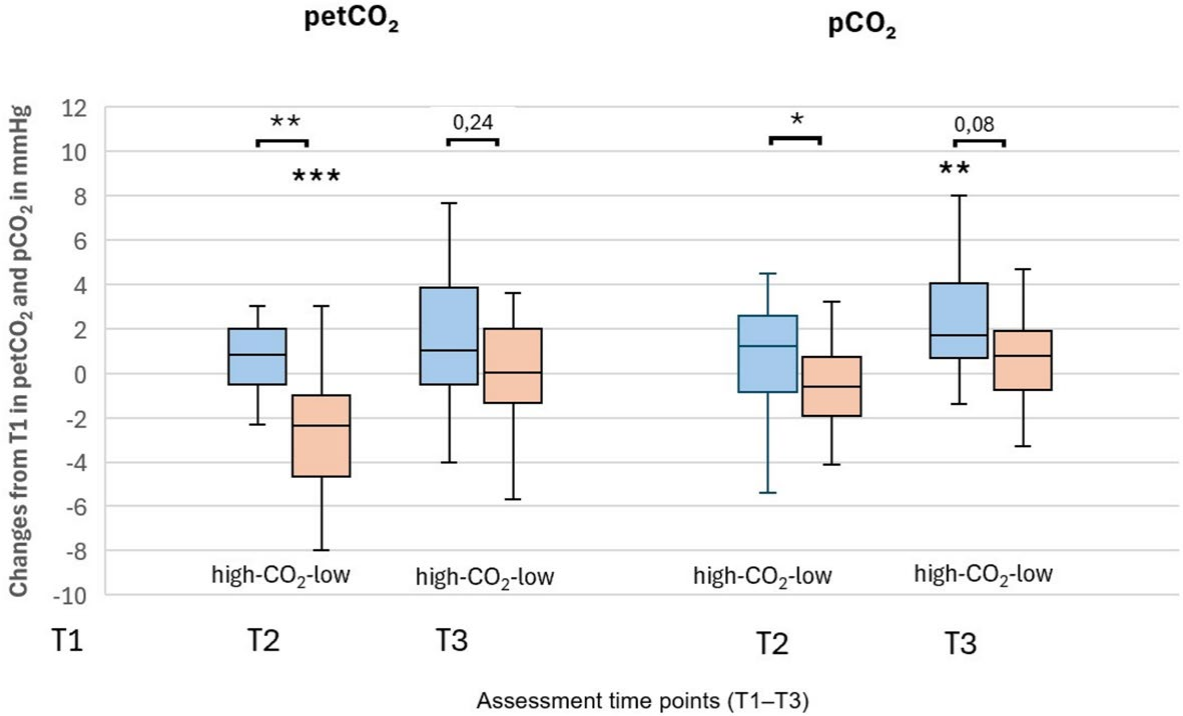
PetCO₂ and PCO₂ changes after speleotherapy in high- versus low-CO₂ therapeutic caves. PetCO₂ (End-Tidal CO₂, mmHg) and pCO₂ (Partial Pressure of CO₂, mmHg). Comparison between intervention groups in therapy caves with high CO₂ (Ennepetal/Bindweide – orange boxes) and therapy caves with low CO₂ (Bodenmais/Prettau – blue boxes). Assessments were conducted at three time points: T1 – before speleotherapy; T2 – immediately after speleotherapy; T3 – three months after speleotherapy. Sample sizes: Ennepetal/Bindweide: T1 – 14, T2 – 14, T3 – 13; Bodenmais/Prettau: T1 – 29, T2 – 27, T3 – 28 (for both PetCO₂ and pCO₂). Wilcoxon test for within-group comparisons across time (*p < 0.05, **p < 0.01, ***p < 0.001); Mann–Whitney U test for between-group comparisons (*p < 0.05, **p < 0.01, ***p < 0.001). Extreme values for PetCO₂ at T2 in the Ennepetal/Bindweide group are omitted for clarity.

- One-day measurement: On a designated therapy day, the PetCO_2_ and respiratory rate were measured immediately before entering the cave, during the therapy session inside the cave, and immediately afterwards. A significant increase in PetCO_2_ was observed during the stay in the cave (*p* = 0.014), accompanied by a significant reduction in the respiratory rate during the therapy session (*p* < 0.001) and immediately after therapy (*p* = 0.034) (data not shown).
- No significant differences were found between caves with higher and lower CO_2_ concentrations.
- Comparison between intervention and control groups:
- In PetCO_2_, pCO_2_ and the respiratory rate no differences were observed between the intervention and the control group; however, a significant reduction in Pet-CO_2_ was found at T2, as well as an increase in pCO_2_ and decrease in the respiratory rate in the intervention group at T3. The NQ score, an indicator of dysfunctional breathing, was significantly lower in the intervention group compared with the control group at T2 and T3 (Additional file 8).
- Comparison of therapy caves with high (Bindweide/Ennepetal) versus therapy caves with low CO_2_ concentrations (Bodenmais/Prettau):
- PetCO_2_ and pCO_2_ were significantly higher at T2 in Bindweide/Ennepetal (*p* < 0.001 and *p* = 0.026, respectively) than in Bodenmais/Prettau. At T3, this difference was no longer significant (p = 0.075). (Fig. 5, Additional file 8).

### Harms

Two participants in the intervention group reported upper respiratory tract infections during the intervention period, which were considered possibly related to the therapy. No serious adverse events were observed.

## Discussion

This is to the best of our knowledge the first randomized controlled study demonstrating that speleotherapy may improve respiratory muscle strength, quality of life parameters in different chronic lung disease and lung function parameters in adult asthmatics. No relevant harms were observed, supporting a favourable benefit–risk profile of speleotherapy as an adjunctive, non-pharmacological intervention.

### Primary endpoint

In patients with asthma, the predefined primary endpoint FeNO remained unchanged, suggesting no measurable effect on type 2 airway inflammation.

### Secondary endpoints and clinical relevance

In contrast, several secondary outcomes improved following speleotherapy. Notably, patient-reported outcomes reached established thresholds for clinically meaningful benefit. The median improvement in asthma control (ACT) met the minimal clinically important difference (MCID) of 3 points *(*^21^ [ACT MCID], and asthma-related quality of life (AQLQ) improved by a median exceeding or meeting the MCID of 0.5 points^22^*)* [AQLQ MCID]. The application of MCID cut-offs to interpret clinically meaningful group-level differences in asthma trials is supported by expert consensus recommendations^23^*)* [MCID consensus].

Physiological outcomes, including lung function parameters and respiratory muscle strength (MIP, MEP), also showed statistically significant changes. However, the magnitude of these changes was modest (e.g., an increase of approximately 4% in FEV₁ and 6–7 cmH₂O in MIP) and may fall within normal biological variation. In addition, clinically meaningful thresholds for these measures are less clearly defined in adult asthma, particularly for lung function parameters reported as percent predicted values. Therefore, these results should be interpreted cautiously and considered supportive of the clinically meaningful benefits observed in ACT and AQLQ. Improvements in Nijmegen Questionnaire scores further suggest reduced dysfunctional breathing symptoms, although no validated MCID is available for this instrument.

Overall, speleotherapy may exert its effects through two complementary mechanisms: improved airway function in asthma and enhanced breathing technique, reflected by better symptom control and quality of life.

Several review articles have addressed the effectiveness of non-pharmacological interventions in bronchial asthma. These approaches include environmental modifications, self-management strategies, physiotherapeutic applications, and multimodal interventions, which may also involve climate-based treatments such as stays at high altitude or by the sea^24,25^. The present findings suggest that speleotherapy could represent an additional supportive option, either as an outpatient intervention or as part of a broader therapeutic setting combining multiple treatment modalities.

The impact of cold and humid environments on chronic respiratory conditions remains a subject of debate^18^. In therapeutic practice, underground climates with temperatures between 5°C and 12°C and relative humidity > 85% has been described in uncontrolled studies as being effective for speleotherapy in patients with asthma^26,27^. As this air is warmed to 37°C within the airways, the relative humidity drops to < 30%, facilitating fluid absorption from the bronchial mucosa and promoting airway clearance. Additionally, the air is free from pollen, pathogens, and spores. High humidity binds dust particles, reducing mucosal irritation^28^.

Another potential mechanism—particularly in asthma—may involve vagal nerve stimulation. The cool environment, exceptional stillness^29^, and unique underground setting probably have vagotonic effects, contributing to reduced respiratory rates and dysfunctional breathing. This aligns with findings from Long COVID patients on the median questionnaire. We therefore hypothesize that, beyond a direct bronchial effect (e.g., reduction in mucosal oedema), indirect effects via vagal activation may play a role.

In Long COVID patients, no significant improvements in lung function were observed; however, signs of improved breathing were noted. The MIP, an indicator of diaphragmatic strength, improved significantly in the intervention group (*p* = 0.048), although this effect did not remain significant after Bonferroni-Holm correction. Improvements were also observed in dyspnoea, “problems with stair climbing/muscle exertion”, anxiety, and sleep disturbances. The reduction in NQ scores supports the hypothesis of improved dysfunctional breathing patterns, although these findings should be interpreted with caution given the small sample size of the Long COVID subgroup. This improvement may also be related to the observed decrease in fatigue severity, as reflected by lower FAS scores. Although FAS scores decreased, the median change of − 3 points remains slightly below the proposed minimal clinically important difference of approximately 4 points for the Fatigue Assessment Scale, indicating a trend toward clinically meaningful improvement but warranting cautious interpretation^30^.

COPD patients reported improvements in CAT scores, despite no measurable improvements in lung function. The observed improvement in CAT scores (e.g., − 2 to − 3 points) reaches or exceeds the established minimal clinically important difference of about 2 points, suggesting that these changes are not only statistically but also clinically meaningful to patients^31^. This is consistent with clinical observations suggesting that COPD patients perceive subjective benefits from speleotherapy even without objective functional findings.

Exposure to the relatively cold environment and moderately elevated CO₂ levels did not result in any serious adverse effects. As reported in the Results section, only minor respiratory infections occurred in a small number of participants. This is consistent with recent evidence from a speleotherapy study in Long COVID patients, which likewise observed no serious adverse events, further supporting the safety of this therapeutic approach^32^.

Although the possibility of response bias in the questionnaire responses due to placebo expectations in the speleotherapy group cannot be fully ruled out, this appears unlikely to have substantially influenced the results, as no significant temporal changes were observed in the SGRQ and in most items of the MEDIAN Long-COVID Questionnaire.

Regarding FeNO values, no significant changes were observed in the overall group or subgroups. This may be due to the high proportion of participants on regular inhaled corticosteroid therapy (overall group: 64%; asthma: 79%).

An elevated CO_2_ concentration in some therapeutic caves may have contributed to bronchial muscle relaxation. In caves with higher CO₂ concentrations, increased levels of CO₂ in the blood and exhaled air were observed, and these elevations partially persisted even three months after treatment (increased SpCO₂), possibly due to altered CO₂ sensitivity. Interestingly, improved dysfunctional breathing (NQ) in the interventional compared to the control group, was associated with higher pCO2 and lower breathing rate at T3 indicating decreased ventilation potentially correcting disease-associated hyperventilation. However, the pattern at T2 (lower pet-CO2 at maintained ventilation) suggest an increase in dead space ventilation.

A further limiting factor for detecting significant effects was the relatively high average age of study participants: total group (62), asthma (61), COPD (69), and Long COVID (55). Among participants under 70, the FEV_1_ improved significantly in the intervention group compared to the control group.

## Limitations

For the asthma group (*n* = 115), the planned sample size of 80 was reached, allowing a statistical power of 0.9.

In the COPD group (*n* = 64), the power was 0.8; in the Long COVID group (*n* = 47), it was 0.7.

Despite randomization, an unequal gender distribution occurred between groups, which may limit the interpretability of results.

All nine participating speleotherapy centers fulfilled the quality criteria outlined in the Introduction, as required for official health resort accreditation (Table 1) (8). However, they differed in terms of patient supervision, the location altitude, radon concentration, and CO₂ content in the cave air.

## Conclusion

Speleotherapy was associated with improvements in lung function and subjective symptoms in asthma patients. However, no significant effect was observed for the primary endpoint (FeNO). Consequently, secondary outcome results should be considered exploratory and require confirmation in future studies.

Furthermore, in the total population (asthma, COPD, Long COVID), an improvement in dysfunctional breathing and a probable reduction in hyperventilation were observed—supported by elevated pCO₂ values. Some of these effects remained measurable 3 months after therapy.

In Long COVID, no objective improvements in lung function were found, but significant improvements were observed in dysfunctional breathing (NQ); fatigue (FAS); and symptoms such as dyspnoea, sleep disturbances, and anxiety.

No improvements in lung function were observed in COPD patients. However, the improvements observed in the CAT questionnaire may support individual reports from COPD patients who described the underground therapy sessions as highly relieving.

It would be desirable for future studies to further investigate the underlying pathophysiological mechanisms of the observed effects—such as the vagus nerve-stimulating influence of speleotherapy on respiratory diseases and stress-related health disorders—since this therapy represents a cost-effective, low-risk, yet long-lasting treatment option.

## Supplementary Information

Supplementary Information 1.

Supplementary Information 2.

Supplementary Information 3.

Supplementary Information 4.

Supplementary Information 5.

Supplementary Information 6.

Supplementary Information 7.

Supplementary Information 8.

Supplementary Information 9.

## Data Availability

The datasets used and analysed during the current study are available from the corresponding author on reasonable request.

## Abbreviations

ACT: Asthma Control Test
AQLQ: Asthma Quality of Life Questionnaire
BMI: Body Mass Index
CAT: COPD Assessment Test
COPD: Chronic Obstructive Pulmonary Disease
DRKS: German Clinical Trials Register
EPOC: EPOC Blood Analysis System (Siemens device)
FAS: Fatigue Assessment Scale
FeNO: Fractional exhaled Nitric Oxide
FEV₁: Forced Expiratory Volume in 1 Second;
FEV₁%: FEV₁ as Percent of Predicted Value
FVC: Forced Vital Capacity
GINA: Global Initiative for Asthma
GOLD: Global Initiative for Chronic Obstructive Lung Disease
ICS: Inhaled Corticosteroids
IQR: Interquartile Range
LABA: Long-Acting Beta-Agonists
LAMA: Long-Acting Muscarinic Antagonists
LC-Median: Median Long COVID Questionnaire
MIP: Maximal Inspiratory Pressure
MEP: Maximal Expiratory Pressure
NQ: Nijmegen Questionnaire
pCO₂: Partial Pressure of Carbon Dioxide in Blood
PEF: Peak Expiratory Flow
PetCO₂: End-Tidal CO₂
PM10: Particulate Matter ≤ 10 Micrometers
RCT: Randomized Controlled Trial
SGRQ: St. George’s Respiratory Questionnaire
SABA: Short-Acting Beta-Agonists
SAMA: Short-Acting Muscarinic Antagonists
SpCO₂: Blood Carbon Dioxide Saturation (measured by spectroanalysis)
SpO₂: Blood Oxygen Saturation (measured by pulse oximetry)
T1/T2/T3: Measurement Time Points: before intervention/after intervention/3 months after intervention
WHO: World Health Organization
WIdO: Scientific Institute of the AOK Health Insurance Fund, Germany
