# Supplementary material for: A randomized controlled trial of adjunctive speleotherapy in asthma, COPD and long COVID
Source: Sci Rep. 2026 May 22;16:15986. doi: 10.1038/s41598-026-52301-4 (PMC13197469; doi:10.1038/s41598-026-52301-4)
Supplement: Supplementary file 1 — Supplementary Information 1. [file 41598_2026_52301_MOESM1_ESM.pdf]

Additional File 1: Assessment Procedures and Measurement Details

Outcome Domain	Measurement / Instrument	Device / Method	Key Procedural Details	Assessor
Airway inflammation (Asthma primary outcome)	Fractional exhaled nitric oxide (FeNO, ppb)	NIOX VERO (NIOX Group)	Measured according to manufacturer guidelines under standardized conditions at T1–T3.	Trained investigators
Lung function (COPD and Long COVID primary outcome, Asthma secondary outcome)	Spirometry: FVC (%), FEV1% (%), FEV1/FVC, PEF (%)	Pneumotrac RMS (Vitalograph)	Spirometry was performed without bronchodilator administration. The highest value from up to three acceptable manoeuvres was used.	Trained investigators
Respiratory muscle strength (COPD and Long COVID primary, Asthma secondary outcome)	Maximal inspiratory pressure (MIP, cmH2O) and maximal expiratory pressure (MEP, cmH2O)	Pneumotrac RMS (Vitalograph)	Participants performed up to three acceptable manoeuvres per test using a flanged mouthpiece. The highest value was used for analysis. MIP was measured from residual volume (RV), and MEP was measured from maximal inspiration (total lung capacity).	Trained investigators
End-tidal CO_2 (CO_2 subproject primary outcome)	PetCO_2 monitoring (mmHg)	Masimo RAD-97	Measured in resting condition. Mean of 3 measurements in 3 minutes	Trained investigators

Outcome Domain	Measurement / Instrument	Device / Method	Key Procedural Details	Assessor
Capillary blood gas analysis (CO_2 subproject secondary outcome)	Capillary pCO_2 (%)	EPOC Blood Analysis System (Siemens)	Capillary blood samples were obtained in resting conditions.	Trained investigators
Asthma symptom control (secondary outcome)	Asthma Control Test (ACT; range 5–25)	5-item questionnaire	Self-administered. Higher scores indicate better asthma control.	Trained investigators
Asthma quality of life (secondary outcome)	Asthma Quality of Life Questionnaire (AQLQ; range 1–7)	32 items, 7-point Likert scale	Measures asthma-related impairment across domains. Higher scores indicate better quality of life.	Trained investigators
COPD symptom burden (secondary outcome)	COPD Assessment Test (CAT; range 0–40)	8 items, 6-point scale	Higher scores indicate greater disease impact.	Trained investigators
Respiratory health-related QoL (COPD secondary outcome)	St. George's Respiratory Questionnaire (SGRQ; range 0–100)	50 items	Higher scores indicate worse health status.	Trained investigators
Dysfunctional breathing (Asthma and COPD secondary outcome; exploratory assessment in COPD)	Nijmegen Questionnaire (NQ; range 0–64)	16 items, cut-off >20	Used in asthma, COPD, and Long COVID groups to assess symptoms of dysfunctional breathing.	Trained investigators
Fatigue (Long COVID secondary outcome)	Fatigue Assessment Scale (FAS; range 10–50)	10 items	Higher scores indicate more severe fatigue.	Trained investigators

Outcome Domain	Measurement / Instrument	Device / Method	Key Procedural Details	Assessor
Long COVID symptom severity (secondary outcome);	The Long-COVID Questionnaire was used with written permission from MEDIAN Klinik Flechtingen (Dr P. O. Schüller, 2023). As this instrument has not been formally published, it is referenced as an unpublished clinical assessment tool. Each item is scored on a scale from 0 to 3; no total score is calculated.	14 items, 4 response categories	Higher scores reflect greater symptom severity.	Trained investigators
Safety outcomes	Adverse events / unintended symptoms	Self-report during sessions	Adverse events were assessed non-systematically through patient self-report during therapy sessions and follow-up visits.	Trained investigators
