# Supplementary material for: A randomized controlled trial of adjunctive speleotherapy in asthma, COPD and long COVID
Source: Sci Rep. 2026 May 22;16:15986. doi: 10.1038/s41598-026-52301-4 (PMC13197469; doi:10.1038/s41598-026-52301-4)
Supplement: Supplementary file 2 — Supplementary Information 2. [file 41598_2026_52301_MOESM2_ESM.pdf]

Additional file 2: Analyzed study participants

Diagnosis	Intervention (98)		Intervention: % per diagnosis	Control (110)		Control: % per diagnosis	Total group(208)		Total group (208)
	female (60 y)	male (66 y)	Total	female (58 y)	male (65 y)	Total	female	male	Total
**Asthma**	42 (62 y)	12 (63 y)	55.10%	37 (58 y)	16 (62 y)	48.20%	79	28	107
**COPD**	6 (62 y)	21 (70 y)	27.60%	7 (69 y)	25 (70 y)	29.10%	13	46	59
**Long-Covid**	12 (53 y)	5 (59 y)	17.30%	18 (52 y)	7 (58 y)	22.70%	30	12	42
**Total group**	60	38	100%	62	48	100%	122	86	208

Additional file 2: Analyzed study participants: intervention group, control group, and total group
