# Supplementary material for: A randomized controlled trial of adjunctive speleotherapy in asthma, COPD and long COVID
Source: Sci Rep. 2026 May 22;16:15986. doi: 10.1038/s41598-026-52301-4 (PMC13197469; doi:10.1038/s41598-026-52301-4)
Supplement: Supplementary file 3 — Supplementary Information 3. [file 41598_2026_52301_MOESM3_ESM.pdf]

Additional file 3: Results for the total study population

Total group		Intervention					Control				Invervention vs control
	p-value Wilcoxon	N	MEDIAN	Range	IQR	p-value Wilcoxon	N	MEDIAN	Range	IQR	p-value U-Test
**Parameter**			50%	[25% ; 75%]				50%	[25% ; 75%]		
**Age** T1		98	65	[57,25 ; 70,75]	13.5		110	64	[55,25 ; 69]	13.75	p = 0,441
**BMI** T1		98	30.02	[25,33 ; 34,29]	8.96		110	28.13	[25,07 ; 32,19]	7.12	p = 0,076
**FeNO** (ppb) T1		95	22	[13 ; 32,5]	19.5		108	24	[15 ; 38]	23	p = 0,470
**FeNO** (ppb) T2-T1	p = 0,797	92	-0.5	[-4,25 ; 5]	9.25	p = 0,884	105	0	[-3 ; 5]	8	p = 0,663
**FeNO** (ppb)T3- T1	p = 0,279	86	-1	[-6 ; 5]	11	p = 0,998	96	-0.5	[-7 ; 7]	14	p = 0,634
**FVC** (%) T1		98	80.13	[69,06 ; 89,51]	20.45		110	82.56	[73,42 ; 90,7]	17.28	p = 0,087
**FVC** (%) T2-T1	**p < 0,001**	96	3.51	[-1,23 ; 8,91]	10.14	**p < 0,001**	109	3.71	[-2,31 ; 7,35]	9.66	p = 0,356
**FVC** (%) T3-T1	**p < 0,001**	89	5.01	[-1,15 ; 9,84]	10.99	**p < 0,001**	101	2.23	[-1,93 ; 7,76]	9.69	p = 0,184
**FEV1** (%) T1		98	74.32	[61,02 ; 88,8]	27.78		110	82.79	[66,46 ; 91,16]	24.7	p = 0,056
**FEV1** (%) T2-T1	**p = 0,002**	96	1.65	[-2,03 ; 5,86]	7.89	**p = 0,003**	109	1.57	[-2,4 ; 5,33]	7.73	p = 0,645
**FEV1** (%) T3-T1	p = 0,415	89	0.56	[-3,95 ; 5,47]	9.42	p = 0,433	101	-0.13	[-2,96 ; 4,29]	7.25	p = 0,980
**FEV1_FVC** T1		98	0.76	[0,66 ; 0,82]	0.16		110	0.79	[0,68 ; 0,83]	0.15	p = 0,135
**FEV1_FVC** T2-T1	**p = 0,040**	96	-0.01	[-0,05 ; 0,02]	0.07	p = 0,029	108	-0.02	[-0,03 ; 0,02]	0.05	p = 0,825
**FEV1_FVC** T3-T1	**p < 0,001**	88	-0.02	[-0,07 ; 0]	0.07	**p < 0,001**	101	-0.03	[-0,06 ; 0,01]	0.07	p = 0,567
**PEF** (%) T1		98	74.5	[60 ; 88,5]	28.5		109	79	[64 ; 91]	27	p = 0,204
**PEF** (%) T2-T1	**p = 0,001**	95	3	[-3 ; 12,5]	15.5	p = 0,054	108	2	[-6,25 ; 12,25]	18.5	p = 0,259
**PEF** (%) T3-T1	**p = 0,035**	89	4	[-5 ; 12]	17	**p = 0,029**	100	1	[-4,5 ; 14,25]	18.75	p = 0,948
**MIP** (cmH2O) T1		98	74	[58,23 ; 91,72]	33.49		109	74.8	[56,3 ; 94,4]	38.1	p = 0,925
**MIP** (cmH2O) T2-T1	**p < 0.001**	96	4.5	[-2,9 ; 12,07]	14.97	p = 0,473	107	-0.3	[-8,2 ; 6,65]	14.85	**p = 0,002**
**MIP** (cmH2O) T3-T1	p = 0,400	89	2.5	[-7,6 ; 7,7]	15.3	p = 0,977	100	-1.05	[-10,67 ; 10,11]	20.78	p = 0,517
**MEP** (cmH2O) T1		97	74.4	[60,6 ; 85,5]	24.9		110	73.88	[55,97 ; 98,15]	42.18	p = 0,874
**MEP** (cmH2O) T2-T1	p = 0,030	94	2.1	[-4,92 ; 15,2]	20.12	p = 0,205	108	-2.35	[-10,45 ; 8,25]	18.7	**p = 0,012**
**MEP** (cmH2O) T3-T1	p = 0,254	86	2.1	[-9,2 ; 16,6]	25.8	p = 0,793	100	0.05	[-9,7 ; 11,96]	21.66	p = 0,359
**NQ** (0-64) T1		98	20	[14 ; 28,5]	14.5		110	23	[15 ; 29]	14	p = 0,261
**NQ** (0-64) T2-T1	**p < 0,001**	98	-3	[-9 ; 1]	10	p = 0,069	109	-1	[-4 ; 3]	7	**p = 0,006**
**NQ** (0-64) T3-T1	**p < 0,001**	95	-2	[-7,5 ; 1]	8.5	p = 0,300	105	0	[-4 ; 3]	7	**p = 0,014**

Additional file 3: Results for the total group regarding baseline results (T1) and the differences (Delta T2-T1, Delta T3-T1) between time points (T1, T2, T3) : FeNO, lung function tests (FVC, FEV_1%, FEV_1/FVC, PEF), respiratory muscle function (MIP, MEP), NQ (Nijmegen Questionnaire); significant results are in **bold** (within-group: $p < 0.025$; between-group: $p < 0.05$).
