# Supplementary material for: A randomized controlled trial of adjunctive speleotherapy in asthma, COPD and long COVID
Source: Sci Rep. 2026 May 22;16:15986. doi: 10.1038/s41598-026-52301-4 (PMC13197469; doi:10.1038/s41598-026-52301-4)
Supplement: Supplementary file 4 — Supplementary Information 4. [file 41598_2026_52301_MOESM4_ESM.pdf]

Additional file 4: Baseline medication

**Total study population**	Total T1 in % (N=number)	Intervention T1 in % (N=number)	Control T1 in % (N=number)
Total study population T1	100% (N=208)	100% (N=98)	100% (N=110)
Respiratory drugs	76.92% (N=160)	83.67% (N=82)	70.9% (N=78)
ICS	63.94% (N=133)	83.67% (N=67)	60% (N=66)
**Asthma group**	Total T1 in % (N=number)	Intervention T1 in % (N=number)	Control T1 in % (N=number)
Asthma group T1	100% (N=107)	100% (N=54)	100% (N=53)
Respiratory drugs	85.05% (N=91)	88.89% (N=48)	81.13% (N=43)
ICS	79.40% (N=85)	81.50% (N=44)	77.40% (N=41)
**COPD group**	Total T1 in % (N=number)	Intervention T1 in % (N=number)	Control T1 in % (N=number)
COPD group T1	100% (N=59)	100% (N=27)	100% (N=32)
Respiratory drugs	88.13% (N=52)	92.59% (N=25)	84.38% (N=27)
ICS	49.20% (N=29)	55.60% (N=15)	53.10% (N=17)
**Long-Covid group**	Total T1 in % (N=number)	Intervention T1 in % (N=number)	Control T1 in % (N=number)
Long-Covid group T1	100% (N=42)	100% (N=17)	100% (N=25)
Respiratory drugs	40.47% (N=17)	52.94% (N=9)	32.00% (N=8)
ICS	38.10% (N=16)	47.10% (N=8)	32.00% (N=8)

Additional file 4: Medication use at time point T1 for the total study population, the asthma group, the COPD group, and the Long Covid group, each divided into total sample, intervention group, and control group. Data are presented as absolute numbers (N) and percentages (%) relative to the respective total sample. No statistical analysis was performed. ICS = inhaled corticosteroids.
