# Supplementary material for: A randomized controlled trial of adjunctive speleotherapy in asthma, COPD and long COVID
Source: Sci Rep. 2026 May 22;16:15986. doi: 10.1038/s41598-026-52301-4 (PMC13197469; doi:10.1038/s41598-026-52301-4)
Supplement: Supplementary file 5 — Supplementary Information 5. [file 41598_2026_52301_MOESM5_ESM.pdf]

Additional file 5: Results for asthma group

Asthma		Intervention					Control				Intervention vs Control	
**Parameter**	**p-value Wilcoxon**	**N**	**MEDIAN**	**Range**	**IQR**	**p-value Wilcoxon**	**N**	**MEDIAN**	**Range**	**IQR**	**p-value U-Test**	**Test; <70 y**
Baseline T1, Delta T2-T1 and Delta T3-T1			**50%**	**[25% ; 75%]**				**50%**	**[25% ; 75%]**			
**Age** T1		54	65	[58 ; 69.75]	11.75		53	61	[55 ; 68]	13	p = 0.224	
**BMI** T1		54	29.81	[24.81 ; 33.71]	8.9		53	27.55	[25.39 ; 31.56]	6.17	p = 0.270	
**FeNO** (ppb) T1		53	24	[13 ; 42]	29		51	25	[16 ; 40]	24	p = 0.656	p = 0.878
**FeNO** (ppb) T2-T1	p = 0.888	50	0	[-4.75 ; 5]	9.75	p = 0.596	49	1	[-3 ; 5]	8	p = 0.947	p = 0.787
**FeNO** (ppb)T3- T1	p = 0.707	51	-1	[-6.5 ; 5]	11.5	p = 0.486	47	-1	[-7 ; 4.5]	11.5	p = 0.644	p = 0.979
**FVC** (%) T1		54	80.92	[71.39 ; 89.96]	18.57		53	84.19	[76.62 ; 90.46]	13.84	p = 0.142	p = 0.084
**FVC** (%) T2-T1	**p < 0.001**	52	5.29	[2.27 ; 9.12]	6.85	**p = 0.004**	52	3.01	[-0.8 ; 6.67]	7.47	**p = 0.010**	**p = 0.011**
**FVC** (%) T3-T1	**p < 0.001**	51	5.98	[-0.5 ; 9.79]	10.29	**p = 0.019**	50	2.49	[-2.02 ; 7.52]	9.54	p = 0.072	p = 0.069
**FEV1** (%) T1		54	81.28	[67.13 ; 90.36]	23.23		53	86.67	[74.6 ; 91.16]	16.56	p = 0.141	p = 0.060
**FEV1** (%) T2-T1	**p < 0.001**	52	3.62	[-1.2 ; 7.64]	8.84	p = 0.174	52	1.38	[-3.22 ; 5.19]	8.41	p = 0.055	**p = 0.035**
**FEV1** (%) T3-T1	p = 0.209	51	0.81	[-3.32 ; 5.88]	9.2	p = 0.923	50	-0.59	[-3.97 ; 4]	7.97	p = 0.335	p = 0.115
**FEV1_FVC** T1		54	0.78	[0.72 ; 0.84]	0.12		53	0.8	[0.76 ; 0.83]	0.07	p = 0.338	p = 0.359
**FEV1_FVC** T2-T1	**p = 0.002**	52	-0.02	[-0.06 ; 0.02]	0.08	p = 0.048	52	-0.02	[-0.03 ; 0.02]	0.05	p = 0.287	p = 0.347
**FEV1_FVC** T3-T1	**p < 0.001**	50	-0.03	[-0.08 ; 0.01]	0.09	**p < 0.001**	50	-0.03	[-0.06 ; 0]	0.06	p = 0.676	p = 0.764
**PEF** (%) T1		54	79	[64 ; 89.75]	25.75		53	85	[70 ; 94]	24	p = 0.138	p = 0.136
**PEF** (%) T2-T1	**p = 0.001**	51	7	[-0.5 ; 13]	13.5	p = 0.937	52	-0.35	[-6.75 ; 8.5]	15.25	**p = 0.016**	**p = 0.010**
**PEF** (%) T3-T1	**p = 0.018**	51	8	[-3 ; 14]	17	p = 0.727	50	0	[-8.93 ; 11.82]	20.75	p = 0.172	p = 0.213
**MIP** (cmH2O) T1		54	72.35	[56.3 ; 90.77]	34.47		53	77.6	[56.3 ; 96]	39.7	p = 0.375	p = 0.999
**MIP** (cmH2O) T2-T1	**p < 0.001**	52	6.5	[0.08 ; 15.53]	15.45	p = 0.226	52	-0.9	[-10.67 ; 5.72]	16.39	**p < 0.001**	**p = 0.002**
**MIP** (cmH2O) T3-T1	**p = 0.020**	51	4.5	[-0.6 ; 11.45]	12.05	p = 0.504	50	-1.6	[-12.62 ; 9]	21.62	**p = 0.030**	p = 0.102
**MEP** (cmH2O) T1		53	66.3	[57 ; 80.1]	23.1		53	70.9	[53.7 ; 92.7]	39	p = 0.297	p = 0.396
**MEP** (cmH2O) T2-T1	**p = 0.013**	50	3.9	[-3.85 ; 17.6]	21.45	p = 0.127	52	-5.2	[-11.8 ; 8.03]	19.83	**p = 0.003**	**p < 0.001**
**MEP** (cmH2O) T3-T1	p = 0.051	50	6.95	[-5.63 ; 19.27]	24.9	p = 0.766	49	-1.8	[-10.3 ; 9.7]	20	p = 0.050	**p = 0.003**
**NQ** (0-64) T1		54	17	[12.25 ; 24.75]	12.5		53	21	[13 ; 28]	15	p = 0.331	p = 0.441
**NQ** (0-64) T2-T1	**p = 0.003**	54	-2.5	[-9 ; 2.75]	11.75	p = 0.380	52	-1	[-4 ; 3]	7	p = 0.066	**p = 0.003**
**NQ** (0-64) T3-T1	**p = 0.001**	54	-3.5	[-7 ; 1.75]	8.75	p = 0.238	52	-1	[-4 ; 2]	6	p = 0.062	**p = 0.021**
**ACT** (5-25) T1		54	17	[14.25 ; 20.75]	6.5		53	18	[14 ; 21]	7	p = 0.522	p = 0.361
**ACT** (5-25) T2-T1	**p < 0.001**	54	3	[1 ; 5]	4	p = 0.751	52	0	[-2 ; 3]	5	**p < 0.001**	**p < 0.001**
**ACT** (5-25) T3-T1	**p = 0.001**	54	1	[0 ; 5]	5	p = 0.224	51	0	[-1 ; 2.5]	3.5	p = 0.091	p = 0.119
**AQLQ** symptoms (1-7) T1		54	4.83	[3.71 ; 5.67]	1.96		53	4.42	[3.67 ; 5.67]	2	p = 0.491	p = 0.613
**AQLQ** symptoms (1-7) T2-T1	**p < 0.001**	54	0.67	[0.19 ; 1.25]	1.06	**p = 0.001**	51	0.42	[0 ; 0.83]	0.83	**p = 0.027**	**p = 0.003**
**AQLQ** symptoms (1-7) T3-T1	**p < 0.001**	53	0.67	[-0.08 ; 1.17]	1.25	**p = 0.002**	51	0.33	[-0.08 ; 0.83]	0.91	p = 0.269	p = 0.181
**AQLQ** activity limitations (1-7) T1		54	4.5	[3.64 ; 5.45]	1.81		53	4.36	[3.91 ; 5.55]	1.64	p = 0.545	p = 0.935
**AQLQ** activity limitations (1-7) T2-T1	**p < 0.001**	54	0.64	[0.11 ; 1.09]	0.98	p = 0.122	51	0.09	[-0.18 ; 0.41]	0.59	**p < 0.001**	**p = 0.001**
**AQLQ** activity limitations (1-7) T3-T1	**p < 0.001**	53	0.45	[0.09 ; 1.18]	1.09	**p = 0.005**	51	0.36	[-0.18 ; 0.7]	0.88	p = 0.087	p = 0.115
**AQLQ** emotional functions (1-7) T1		54	4.9	[4 ; 6.1]	2.1		53	4.6	[3.8 ; 5.8]	2	p = 0.504	p = 0.497
**AQLQ** emotional functions (1-7) T2-T1	**p < 0.001**	53	0.6	[0.2 ; 1.2]	1	**p = 0.001**	51	0.2	[0 ; 0.8]	0.8	p = 0.091	**p = 0.031**
**AQLQ** emotional functions (1-7) T3-T1	**p = 0.004**	53	0.6	[-0.4 ; 1]	1.4	**p < 0.001**	51	0.4	[-0.1 ; 1]	1.1	p = 0.909	p = 0.696

**AQLQ** environmental exposure (1-7) T1		54	5	[3.75 ; 5.75]	2		53	4.75	[3.5 ; 5.75]	2.25	p = 0.537	p = 0.232
**AQLQ** environmental exposure (1-7) T2-T1	p < 0.001	54	0.75	[0.06 ; 1.44]	1.38	p = 0.018	51	0.25	[-0.12 ; 1]	1.12	p = 0.048	p = 0.173
**AQLQ** environmental exposure (1-7) T3-T1	p = 0.032	53	0.5	[-0.5 ; 1]	1.5	p = 0.076	51	0.25	[-0.25 ; 0.75]	1	p = 0.544	p = 0.546
**AQLQ** total score (1-7) T1		54	4.67	[3.73 ; 5.52]	1.79		53	4.28	[3.78 ; 5.62]	1.84	p = 0.720	p = 0.658
**AQLQ** total score (1-7) T2-T1	p < 0.001	54	0.69	[0.17 ; 1.26]	1.09	p < 0.001	51	0.28	[0.02 ; 0.66]	0.64	p = 0.007	p = 0.005
**AQLQ** total score (1-7) T3-T1	p < 0.001	53	0.47	[0.03 ; 1.16]	1.13	p < 0.001	51	0.31	[-0.06 ; 0.72]	0.78	p = 0.224	p = 0.202

Additional file 5: Results for the asthma group regarding baseline results (T1) and the differences (Delta T2-T1, Delta T3-T1) between time points (T1, T2, T3): FeNO, lung function (FVC, FEV_1%, FEV_1/FVC, PEF), respiratory muscle tests (MIP, MEP), NQ (Nijmegen Questionnaire), ACT (Asthma-Control-Test), AQLQ (Asthma Quality of Life Questionaire). Significant results in **bold** (within-group: $p < 0.025$; between-group: $p < 0.05$)
