# Supplementary material for: A randomized controlled trial of adjunctive speleotherapy in asthma, COPD and long COVID
Source: Sci Rep. 2026 May 22;16:15986. doi: 10.1038/s41598-026-52301-4 (PMC13197469; doi:10.1038/s41598-026-52301-4)
Supplement: Supplementary file 6 — Supplementary Information 6. [file 41598_2026_52301_MOESM6_ESM.pdf]

Additional file 6: Results for COPD group

**COPD**		**Intervention**					**Control**				**Intervention vs control**
**Parameter**	**p-value Wilcoxon**	**N**	**MEDIAN**	**Range**	**IQR**	**p-value Wilcoxon**	**N**	**MEDIAN**	**Range**	**IQR**	**p-value U-Test**
Baseline T1, Delta T2-T1 and Delta T3-T1		**CI =**	**50%**	**[25% ; 75%]**			**CK =**	**50%**	**[25% ; 75%]**		
**Age** T1		27	67	[64.5 ; 73]	8.5		32	69	[66 ; 73]	7	p = 0.310
**BMI** T1		27	30.09	[27.93 ; 33.57]	5.64		32	29.29	[25.39 ; 32.38]	6.99	p = 0.338
**FeNO** (ppb) T1		26	21	[15 ; 25.75]	10.75		32	23.5	[15.25 ; 30]	14.75	p = 0.247
**FeNO** (ppb) T2-T1	p = 0.664	26	-1	[-5.5 ; 3]	8.5	p = 0.155	32	-1	[-7.25 ; 3.25]	10.5	p = 0.802
**FeNO** (ppb)T3- T1	p = 0.457	22	-2.5	[-4.75 ; 4.25]	9	p = 0.817	28	-1	[-9.25 ; 8.25]	17.5	p = 0.860
**FVC** (%) T1		27	70	[58.28 ; 81.37]	23.09		32	73.8	[62.39 ; 82.81]	20.42	p = 0.346
**FVC** (%) T2-T1	p = 0.824	27	1.42	[-5.35 ; 9.35]	14.7	**p = 0.013**	32	6.06	[-4.57 ; 8.57]	13.14	p = 0.403
**FVC** (%) T3-T1	p = 0.035	23	7.77	[0.14 ; 11.39]	11.25	**p = 0.001**	29	4.95	[-0.89 ; 10.9]	11.79	p = 0.678
**FEV1** (%) T1		27	49.31	[42.81 ; 67.16]	24.35		32	62.61	[51.39 ; 77.97]	26.58	p = 0.026
**FEV1** (%) T2-T1	p = 0.522	27	0.75	[-1.78 ; 4.41]	6.19	p = 0.295	32	0.57	[-2.44 ; 4.38]	6.82	p = 0.915
**FEV1** (%) T3-T1	p = 0.501	23	2.47	[-2.56 ; 5.81]	8.37	p = 0.717	29	-0.13	[-2.33 ; 5.15]	7.48	p = 0.638
**FEV1_FVC** T1		27	0.59	[0.51 ; 0.71]	0.2		32	0.68	[0.62 ; 0.78]	0.16	p = 0.025
**FEV1_FVC** T2-T1	p = 0.549	27	-0.02	[-0.06 ; 0.02]	0.08	p = 0.051	32	-0.02	[-0.05 ; 0.01]	0.06	p = 0.737
**FEV1_FVC** T3-T1	**p = 0.001**	23	-0.02	[-0.06 ; -0.01]	0.05	**p = 0.001**	29	-0.03	[-0.07 ; 0]	0.07	p = 0.934
**PEF** (%) T1		27	60	[45.5 ; 76]	30.5		31	65	[58 ; 81]	23	p = 0.165
**PEF** (%) T2-T1	p = 0.956	27	1	[-3.5 ; 4]	7.5	p = 0.136	31	3	[-6 ; 11.5]	17.5	p = 0.399
**PEF** (%) T3-T1	p = 0.953	23	0	[-5 ; 5]	10	p = 0.193	28	0	[-3.25 ; 9.5]	12.75	p = 0.399
**MIP** (cmH2O) T1		27	81.8	[62.8 ; 93.1]	30.3		31	75.2	[58.55 ; 92.2]	33.65	p = 0.366
**MIP** (cmH2O) T2-T1	p = 0.703	27	0.4	[-7.4 ; 7.05]	14.45	p = 0.831	31	1.7	[-7.15 ; 5.75]	12.9	p = 0.882
**MIP** (cmH2O) T3-T1	p = 0.142	23	-1.9	[-10.1 ; 2.65]	12.75	p = 0.330	28	4.6	[-4.6 ; 9]	13.6	p = 0.062
**MEP** (cmH2O) T1		27	93.1	[73.45 ; 110.75]	37.3		32	78.45	[64.62 ; 101.12]	36.5	p = 0.244
**MEP** (cmH2O) T2-T1	p = 0.272	27	2.2	[-2.05 ; 19.85]	21.9	p = 0.815	32	-1.5	[-10.1 ; 10.89]	20.99	p = 0.148
**MEP** (cmH2O) T3-T1	p = 0.545	22	1.5	[-7.7 ; 12.23]	19.93	p = 0.674	29	-1.1	[-10.5 ; 13.6]	24.1	p = 0.909
**NQ** (0-64) T1		27	17	[14 ; 25]	11		32	20	[10.75 ; 27]	16.25	p = 0.659
**NQ** (0-64) T2-T1	p = 0.065	27	-2	[-6 ; 0.5]	6.5	p = 0.592	32	-1.5	[-3.25 ; 2.25]	5.5	p = 0.333
**NQ** (0-64) T3-T1	p = 0.260	25	-1	[-5 ; 1]	6	p = 0.744	29	0	[-2 ; 3]	5	p = 0.305
**CAT** (0-40) T1		26	19	[14 ; 24]	10		31	20	[16 ; 22.5]	6.5	p = 0.923
**CAT** (0-40) T2-T1	**p = 0.019**	26	-2	[-5.75 ; 0]	5.75	p = 0.827	31	1	[-3 ; 3]	6	**p = 0.036**
**CAT** (0-40) T3-T1	p = 0.076	24	-3	[-4.25 ; 0]	4.25	p = 0.640	28	-1	[-3.5 ; 3]	6.5	p = 0.306

**SGRQ** symptoms (0-100) T1		26	51.75	[32.2 ; 69.25]	37.05		31	62.16	[49.53 ; 69.22]	19.69	p = 0.383
**SGRQ** symptoms (0-100) T2-T1	p = 0.098	26	2.49	[-3.56 ; 9.78]	13.34	p = 0.120	30	-3.76	[-10.51 ; 2.71]	13.22	p = 0.051
**SGRQ** symptoms (0-100) T3-T1	p = 0.491	24	-2.48	[-7.44 ; 7.35]	14.79	p = 0.117	29	-3.64	[-16.27 ; 3.35]	19.62	p = 0.453
**SGRQ** activity (0-100) T1		26	59.46	[49.18 ; 70.8]	21.62		31	60.04	[53.62 ; 72.29]	18.67	p = 0.841
**SGRQ** activity (0-100) T2-T1	p = 0.290	26	-4.93	[-11.66 ; 4.88]	16.54	p = 0.093	30	-0.7	[-6.82 ; 5.22]	12.04	p = 0.941
**SGRQ** activity (0-100) T3-T1	p = 0.294	24	-0.93	[-8 ; 5.86]	13.86	p = 0.010	29	-6.13	[-12.83 ; 0]	12.83	p = 0.502
**SGRQ** impacts (0-100) T1		26	33.42	[19.65 ; 38.43]	18.78		31	32.76	[20.84 ; 43.38]	22.54	p = 0.522
**SGRQ** impacts (0-100) T2-T1	p = 0.305	26	0	[-7.66 ; 4.41]	12.07	p = 0.123	30	-2.21	[-9.42 ; 1.34]	10.76	p = 0.511
**SGRQ** impacts (0-100) T3-T1	p = 0.045	24	-4.75	[-7.84 ; 0.5]	8.34	p = 0.036	29	-3.5	[-10.35 ; -0.16]	10.19	p = 0.775
**SGRQ** total score (0-100) T1		26	46.4	[35.7 ; 49.36]	13.66		31	43.88	[34.78 ; 55.52]	20.74	p = 0.532
**SGRQ t**otal score (0-100) T2-T1	p = 0.922	26	0.76	[-4.46 ; 3.77]	8.23	p = 0.036	30	-3.02	[-7.37 ; 1.62]	8.99	p = 0.148
**SGRQ t**otal score (0-100) T3-T1	p = 0.065	24	-2.14	[-7.1 ; 1.11]	8.21	p = 0.002	29	-3.37	[-8.28 ; -1.85]	6.43	p = 0.313

Additional file 6: Results for the COPD group regarding baseline results (T1) and the differences (Delta T2-T1, Delta T3-T1) between time points (T1, T2, T3): Lung function (FVC, FEV_1%, FEV_1/FVC, PEF), respiratory muscle tests (MIP, MEP), NQ (Nijmegen Questionnaire), CAT (COPD Assessment Test), SGRQ (St. George's Respiratory Questionnaire). Significant results in bold (within-group: $p < 0.025$; between-group: $p < 0.05$)
