# Supplementary material for: A randomized controlled trial of adjunctive speleotherapy in asthma, COPD and long COVID
Source: Sci Rep. 2026 May 22;16:15986. doi: 10.1038/s41598-026-52301-4 (PMC13197469; doi:10.1038/s41598-026-52301-4)
Supplement: Supplementary file 7 — Supplementary Information 7. [file 41598_2026_52301_MOESM7_ESM.pdf]

Additional file 7: Results for Long-Covid group

Long Covid		Intervention					Control				Intervention vs Control
**Parameter**	**p-value Wilcoxon**	**N**	**MEDIAN**	**Range**	**IQR**	**p-value Wilcoxon**	**N**	**MEDIAN**	**Range**	**IQR**	**p-value U-Test**
Baseline T1, Delta T2-T1 and Delta T3-T1			**50%**	**[25% ; 75%]**				**50%**	**[25% ; 75%]**		
**Age** T1		17	56	[52 ; 60]	8		25	55	[46 ; 62]	16	p = 0.918
**BMI** T1		17	32.59	[24.47 ; 37.63]	13.16		25	28.48	[23.41 ; 32.27]	8.86	p = 0.405
**FeNO** (ppb) T1		16	23	[12 ; 26.75]	14.75		25	22	[12 ; 30]	18	p = 0.936
**FeNO** (ppb) T2-T1	p = 0.542	16	-2	[-2.25 ; 6]	8.25	p = 0.258	24	0.5	[-1.25 ; 6]	7.25	p = 0.353
**FeNO** (ppb)T3- T1	p = 0.314	13	-1	[-6 ; 1]	7	p = 0.167	21	3	[-2 ; 13]	15	p = 0.082
**FVC** (%) T1		17	89.34	[76.76 ; 93.36]	16.6		25	88.55	[80.31 ; 98.58]	18.27	p = 0.465
**FVC** (%) T2-T1	p = 0.599	17	0.31	[-6.27 ; 3.67]	9.94	p = 0.074	25	1.01	[-3.41 ; 7.59]	11	p = 0.481
**FVC** (%) T3-T1	p = 0.762	15	-1.27	[-4.08 ; 4.26]	8.34	p = 0.924	22	0.24	[-2.81 ; 5.38]	8.19	p = 0.643
**FEV1** (%) T1		17	90	[77.56 ; 95.39]	17.83		25	89.43	[80.47 ; 95.99]	15.52	p = 0.990
**FEV1** (%) T2-T1	p = 0.847	17	0.73	[-2.89 ; 2.25]	5.14	**p = 0.003**	25	4.14	[0 ; 9.09]	9.09	p = 0.109
**FEV1** (%) T3-T1	p = 0.121	15	-2.86	[-5.42 ; 0.1]	5.52	p = 0.198	22	1.73	[-2.06 ; 5.48]	7.54	**p = 0.038**
**FEV1_FVC** T1		17	0.81	[0.78 ; 0.85]	0.07		25	0.81	[0.72 ; 0.86]	0.14	p = 0.807
**FEV1_FVC** T2-T1	p = 0.337	17	0.02	[-0.01 ; 0.04]	0.05	p = 0.748	24	-0.01	[-0.02 ; 0.03]	0.05	p = 0.346
**FEV1_FVC** T3-T1	p = 0.279	15	-0.02	[-0.05 ; 0.01]	0.06	p = 0.639	22	-0.01	[-0.04 ; 0.06]	0.1	p = 0.733
**PEF** (%) T1		17	80	[70 ; 96]	26		25	76	[63 ; 89]	26	p = 0.663
**PEF** (%) T2-T1	p = 0.123	17	2	[0 ; 13]	13	p = 0.038	25	9	[-5 ; 16.1]	21.1	p = 0.617
**PEF** (%) T3-T1	p = 0.703	15	3	[-8.5 ; 10]	18.5	**p = 0.009**	22	10.55	[-1.5 ; 18.25]	19.75	p = 0.133
**MIP** (cmH2O) T1		17	72.2	[54.3 ; 84.2]	29.9		25	67.9	[50.3 ; 92]	41.7	p = 0.788
**MIP** (cmH2O) T2-T1	p = 0.048	17	6.4	[-0.9 ; 12]	12.9	p = 0.812	24	0.75	[-2.5 ; 11.03]	13.53	p = 0.397
**MIP** (cmH2O) T3-T1	p = 0.715	15	-2.9	[-12.2 ; 7.15]	19.35	p = 0.993	22	-2.85	[-8.3 ; 13.78]	22.08	p = 0.578
**MEP** (cmH2O) T1		17	78.4	[64.4 ; 87.1]	22.7		25	73.3	[53.6 ; 104.7]	51.1	p = 0.838
**MEP** (cmH2O) T2-T1	p = 0.670	17	-4.4	[-8 ; 1.9]	9.9	p = 0.740	24	1.85	[-4.8 ; 8.25]	13.05	p = 0.204
**MEP** (cmH2O) T3-T1	p = 0.104	14	-8.05	[-20.9 ; 2.98]	23.88	p = 0.591	22	2.8	[-4.43 ; 10.9]	15.33	p = 0.085
**NQ** (0-64) T1		17	30	[24 ; 34]	10		25	29	[24 ; 33]	9	p = 0.908
**NQ** (0-64) T2-T1	**p < 0.001**	17	-8	[-10 ; -3]	7	p = 0.095	25	-2	[-6 ; 1]	7	**p = 0.014**
**NQ** (0-64) T3-T1	p = 0.066	16	-6.5	[-9.25 ; 0.25]	9.5	p = 0.494	24	1	[-5 ; 2.5]	7.5	p = 0.193
**FAS** (0-40) T1		17	26	[23 ; 32]	9		25	28	[23 ; 33]	10	
**FAS** (0-40) T2-T1	p = 0.268	17	-1	[-3 ; 1]	4	p = 0.636	23	0	[-4 ; 1.5]	5.5	p = 0.711
**FAS** (0-40) T3-T1	**p = 0.011**	16	-3	[-6.5 ; 0]	6.5	p = 0.256	22	-1	[-3.75 ; 2]	5.75	p = 0.177
**SGRQ** symptoms (0-100) T1		17	55.44	[47.25 ; 66.4]	19.15		23	63.61	[37.24 ; 74.6]	37.36	p = 0.420
**SGRQ** symptoms (0-100) T2-T1	p = 0.359	17	-3.29	[-9.93 ; 4.12]	14.05	p = 0.548	22	-1.05	[-9.64 ; 4.52]	14.16	p = 0.832
**SGRQ** symptoms (0-100) T3-T1	p = 0.348	16	-4.13	[-12.02 ; 8.93]	20.95	p = 0.523	21	-4.9	[-8.42 ; 5.39]	13.81	p = 0.878
**SGRQ** activity (0-100) T1		17	59.46	[53.53 ; 72.33]	18.8		23	59.46	[44.92 ; 82.72]	37.8	p = 0.967
**SGRQ** activity (0-100) T2-T1	p = 0.934	17	1.84	[-6.02 ; 6.14]	12.16	p = 0.223	22	0	[-6.51 ; 0.96]	7.47	p = 0.452
**SGRQ** activity (0-100) T3-T1	p = 0.389	16	-3.96	[-8.25 ; 6.05]	14.3	p = 0.911	21	0	[-6.7 ; 6.63]	13.33	p = 0.480
**SGRQ** impacts (0-100) T1		17	35.47	[30.53 ; 39.68]	9.15		23	36.79	[15.61 ; 53.62]	38.01	p = 0.859
**SGRQ** impacts (0-100) T2-T1	p = 0.495	17	4.5	[-3.83 ; 9.57]	13.4	p = 0.596	22	-1.29	[-11.67 ; 6.46]	18.13	p = 0.257
**SGRQ** impacts (0-100) T3-T1	p = 0.632	16	-0.91	[-10.92 ; 8.14]	19.06	p = 0.729	21	1.56	[-7.98 ; 11.04]	19.02	p = 0.602

**SGRQ** total score (0-100) T1		17	46.22	[43.43 ; 50.37]	6.94		23	48.5	[31.11 ; 66.45]	35.34	p = 0.774
**SGRQ t**otal score (0-100) T2-T1	p = 0.782	17	1.26	[-3.85 ; 6.13]	9.98	p = 0.498	22	0.27	[-7.76 ; 4.72]	12.48	p = 0.533
**SGRQ t**otal score (0-100) T3-T1	p = 0.375	16	-1.3	[-12.06 ; 4.69]	16.75	p = 0.729	21	-0.13	[-7.42 ; 8.67]	16.09	p = 0.374
**LC-Median** dyspnea after exercise (0-3) T1		17	3	[2 ; 3]	1		25	2	[1 ; 3]	2	**p = 0.040**
**LC-Median** dyspnea after exercise (0-3) T2-T1	p = 0.219	17	0	[-1 ; 0]	1	p = 0.056	25	0	[0 ; 1]	1	**p = 0.026**
**LC-Median** dyspnea after exercise (0-3) T3-T1	**p = 0.004**	15	-1	[-1 ; 0]	1	p = 0.183	23	0	[0 ; 1]	1	**p = 0.001**
**LC-Median** problems with stair climbing and muscle exertion (0-3) T1		17	3	[2 ; 3]	1		25	2	[2 ; 3]	1	p = 0.077
**LC-Median** problems with stair climbing and muscle exertion (0-3) T2-T1	p = 0.125	17	0	[0 ; 0]	0	p = 0.531	25	0	[0 ; 0]	0	**p = 0.042**
**LC-Median** problems with stair climbing and muscle exertion (0-3) T3-T1	p = 0.109	15	0	[-1 ; 0]	1	p = 0.555	23	0	[0 ; 0]	0	p = 0.085
**LC-Median** coughing (0-3) T1		17	1	[1 ; 2]	1		25	1	[0 ; 2]	2	p = 0.905
**LC-Median** coughing (0-3) T2-T1	p = 0.453	17	0	[-1 ; 0]	1	p = 0.469	25	0	[0 ; 0]	0	p = 0.111
**LC-Median** coughing (0-3) T3-T1	p = 0.359	15	0	[-1 ; 0]	1	p = 0.555	23	0	[0 ; 0]	0	p = 0.386
**LC-Median** palpitation (0-3) T1		17	2	[1 ; 2]	1		25	1	[1 ; 2]	1	p = 0.786
**LC-Median** palpitation (0-3) T2-T1	p = 0.999	17	0	[0 ; 0]	0	p = 0.781	25	0	[0 ; 0]	0	p = 0.738
**LC-Median** palpitation (0-3) T3-T1	p = 0.999	15	0	[0 ; 0]	0	p = 0.999	23	0	[-1 ; 0.5]	1.5	p = 0.756
**LC-Median** fatigue (0-3) T1		17	3	[2 ; 3]	1		25	3	[2 ; 3]	1	p = 0.987
**LC-Median** fatigue (0-3) T2-T1	p = 0.250	17	0	[0 ; 0]	0	p = 0.999	25	0	[0 ; 0]	0	p = 0.452
**LC-Median** fatigue (0-3) T3-T1	p = 0.250	15	0	[0 ; 0]	0	p = 0.999	23	0	[0 ; 0]	0	p = 0.383
**LC-Median** joint and muscle pain (0-3) T1		17	2	[1 ; 3]	2		25	2	[2 ; 3]	1	p = 0.693
**LC-Median** joint and muscle pain (0-3) T2-T1	p = 0.453	17	0	[-1 ; 0]	1	p = 0.999	25	0	[0 ; 0]	0	p = 0.539
**LC-Median** joint and muscle pain (0-3) T3-T1	p = 0.625	15	0	[0 ; 0]	0	p = 0.999	23	0	[-0.5 ; 0]	0.5	p = 0.305
**LC-Median** olfactory and gustatory disorders (0-3) T1		17	1	[0 ; 1]	1		25	1	[0 ; 1]	1	p = 0.945
**LC-Median** olfactory and gustatory disorders (0-3) T2-T1	p = 0.531	17	0	[0 ; 0]	0	p = 0.625	25	0	[0 ; 0]	0	p = 0.226
**LC-Median** olfactory and gustatory disorders (0-3) T3-T1	p = 0.500	15	0	[0 ; 0]	0	p = 0.999	23	0	[0 ; 0]	0	p = 0.134
**LC-Median** balance and fine motor imbalace (0-3) T1		17	1	[1 ; 2]	1		25	1	[1 ; 2]	1	p = 0.979
**LC-Median** balance and fine motor imbalace (0-3) T2-T1	p = 0.999	17	0	[0 ; 0]	0	p = 0.359	25	0	[0 ; 0]	0	p = 0.461
**LC-Median** balance and fine motor imbalace (0-3) T3-T1	p = 0.398	15	0	[0 ; 1]	1	p = 0.270	23	0	[0 ; 1]	1	p = 0.773
**LC-Median** word-finding difficulties (0-3) T1		17	3	[2 ; 3]	1		25	2	[1 ; 2]	1	p = 0.091
**LC-Median** word-finding difficulties (0-3) T2-T1	p = 0.625	17	0	[0 ; 0]	0	p = 0.999	25	0	[0 ; 0]	0	p = 0.514
**LC-Median** word-finding difficulties (0-3) T3-T1	p = 0.625	15	0	[0 ; 0]	0	p = 0.766	23	0	[0 ; 0]	0	p = 0.321
**LC-Median** problems with concentration and verbal indurance (0-3) T1		17	2	[2 ; 3]	1		25	2	[2 ; 3]	1	p = 0.526
**LC-Median** problems with concentration and verbal indurance (0-3) T2-T1	p = 0.250	17	0	[0 ; 0]	0	p = 0.432	25	0	[-1 ; 0]	1	p = 0.904
**LC-Median** problems with concentration and verbal indurance (0-3) T3-T1	p = 0.625	15	0	[0 ; 0]	0	p = 0.875	23	0	[0 ; 0]	0	p = 0.591
**LC-Median** Anxiety and sleep disorders (0-3) T1		17	2	[2 ; 3]	1		25	2	[1 ; 2]	1	p = 0.276
**LC-Median** Anxiety and sleep disorders (0-3) T2-T1	p = 0.125	17	0	[-1 ; 0]	1	p = 0.375	25	0	[0 ; 0]	0	**p = 0.021**
**LC-Median** Anxiety and sleep disorders (0-3) T3-T1	p = 0.359	15	0	[-1 ; 0]	1	p = 0.234	22	0	[0 ; 0.75]	0.75	p = 0.068
**LC-Median** lack of interest, loneliness (0-3) T1		17	1	[0 ; 3]	3		25	2	[1 ; 2]	1	p = 0.811
**LC-Median** lack of interest, loneliness (0-3) T2-T1	p = 0.999	17	0	[0 ; 0]	0	p = 0.999	25	0	[0 ; 0]	0	p = 0.999
**LC-Median** lack of interest, loneliness (0-3) T3-T1	p = 0.562	15	0	[0 ; 1]	1	p = 0.999	23	0	[-0.5 ; 0.5]	1	p = 0.520
**LC-Median** problems controlling emotions (0-3) T1		17	1	[1 ; 2]	1		25	1	[0 ; 2]	2	p = 0.915
**LC-Median** problems controlling emotions (0-3) T2-T1	p = 0.500	17	0	[0 ; 0]	0	p = 0.754	25	0	[0 ; 0]	0	p = 0.387
**LC-Median** problems controlling emotions (0-3) T3-T1	p = 0.999	15	0	[-0.5 ; 0]	0.5	p = 0.807	23	0	[0 ; 1]	1	p = 0.449

**LC-Median** psychotrauma caused by Covid 19 (0-3) T1		17	1	[0 ; 2]	2		25	1	[0 ; 2]	2	p = 0.873
**LC-Median** psychotrauma caused by Covid 19 (0-3) T2-T1	p = 0.375	17	0	[-1 ; 0]	1	p = 0.999	25	0	[0 ; 0]	0	p = 0.085
**LC-Median** psychotrauma caused by Covid 19 (0-3) T3-T1	p = 0.750	15	0	[0 ; 0]	0	p = 0.688	23	0	[0 ; 0]	0	p = 0.325

Additional file 7: Results for Long Covid group regarding baseline results (T1) and the differences (Delta T2-T1, Delta T3-T1) between time points (T1, T2, T3): FeNO, lung function (FVC, FEV_1%, FEV_1/FVC, PEF), respiratory muscle tests (MIP, MEP), NQ (Nijmegen Questionnaire), FAS (Fatigue Assessment Scale), SGRQ (St. George’s Respiratory Questionnaire), LC-Median (Median Long COVID Questionnaire). Significant results in **bold** (within-group: $p < 0.025$; between-group: $p < 0.05$)
