# Supplementary material for: A randomized controlled trial of adjunctive speleotherapy in asthma, COPD and long COVID
Source: Sci Rep. 2026 May 22;16:15986. doi: 10.1038/s41598-026-52301-4 (PMC13197469; doi:10.1038/s41598-026-52301-4)
Supplement: Supplementary file 8 — Supplementary Information 8. [file 41598_2026_52301_MOESM8_ESM.pdf]

Additional file 8: Respiratory parameters (petCO_2, pCO_2, pH, respiratory rate, NQ) and CO_2- concentration in the speleotherapy cave (sample size indicated in paratheses)

**Pet-CO2 (mmHg)**	Total group				Intervention group (elevated CO2 (Ennepetal, Bindweide) / low CO2 (Prettau-Bodenmais))			
	Intervention	Control	p (within group)	p (between groups)	Intervention group elevated CO2 (H)	Intervention group low CO2 (L)	p (within group)	p (between groups)
T1	32.46 (51)	31.91 (56)			32.26 (14)	32.63 (29)		
T2	31.33* (49)	32.11 (56)	0.031 (I)		33.4 (14)	29.89*(26)	**<0.001 (L)**	**<0.001**
T3	33.11 (49)	32.59 (49)			34.02 (13)	32.64 (28)		

**pCO2 (mmHg)**	Total group				Intervention group (elevated CO2 (Ennepetal. Bindweide) / low CO2 (Prettau-Bodenmais))			
	Intervention	Control	p (within group)	p (between groups)	Intervention group elevated CO2 (H)	Intervention group low CO2 (L)	p (within group)	p (between groups)
T1	33.88 (49)	33.62 (56)			33.44 (14)	34.22 (29)		
T2	33.52 (49)	33.58 (56)			34.34 (14)	33.47 (26)		0.026
T3	34.84* (47)	33.98 (48)	**0.012 (I)**		35.85* (13)	34.8 (28)	**0.005 (H)**	

**pH**	Total group				Intervention group (elevated CO2 (Ennepetal. Bindweide) / low CO2 (Prettau-Bodenmais))			
	Intervention	Control	p (within group)	p (between groups)	Intervention group elevated CO2 (H)	Intervention group low CO2 (L)	p (within group)	p (between groups)
T1	7.44 (51)	7.43 (56)			7.45 (14)	7.43 (29)		
T2	7.44 (49)	7.44 (56)			7.44 (14)	7.44 (26)		
T3	7.43 (49)	7.44 (48)			7.43 (13)	7.43 (28)		

**Respiratory rate (breaths/min)**	Total group				Intervention group (elevated CO2 (Ennepetal. Bindweide) / low CO2 (Prettau-Bodenmais))			
	Intervention	Control	p (within group)	p (between groups)	Intervention group elevated CO2 (H)	Intervention group low CO2 (L)	p (within group)	p (between groups)
T1	15.57 (51)	14.9 (56)			14.54 (14)	16.19 (29)		
T2	15.29 (49)	14.91 (56)			14.49 (14)	15.31 (26)		
T3	14.92* (49)	14.88 (49)	0.032 (I)		13.67 (13)	15.64 (28)		

**NQ (16 items, cut-off >20, max 64)**	Total group				Intervention group (elevated CO2 (Ennepetal. Bindweide) / low CO2 (Prettau-Bodenmais))			
	Intervention	Control	p (within group)	p (between groups)	Intervention group elevated CO2 (H)	Intervention group low CO2 (L)	p (within group)	p (between groups)
T1	21.24 (96)	22.19 (109)			20 (14)	22.03 (29)		
T2	17.27* (96)	21.08 (108)	**<0.001 (I)**	0.007	18.43 (14)	16.97* (26)	**0.008 (L)**	
T3	18.3* (93)	21.52 (104)	**<0.001 (I)**	0.017	19.92 (13)	17.86* (28)	**0.004 (L)**	

Additional file 8: Overview of the following parameters: PetCO_2 (End-Tidal CO_2), pCO_2 (Partial Pressure of CO2), pH, respiratory rate, NQ (Nijmegen Questionnaire)
Comparisons across the total sample (intervention vs. control) and between caves with elevated CO_2 (H) versus low CO_2 (L) concentrations
✶: significant within group, Sample size indicated in parentheses, Significant values are highlighted in **bold** numbers(group noted in parentheses: I = intervention, C = control)
