# Supplementary material for: A randomized controlled trial of adjunctive speleotherapy in asthma, COPD and long COVID
Source: Sci Rep. 2026 May 22;16:15986. doi: 10.1038/s41598-026-52301-4 (PMC13197469; doi:10.1038/s41598-026-52301-4)
Supplement: Supplementary file 9 — Supplementary Information 9. [file 41598_2026_52301_MOESM9_ESM.pdf]

Additional File 9

Table 2: Longitudinal data analysis by Brunner and Langer

Disease	Parameter	Group	Time	Group x Time
**Athma**		p-values	p-values	p-values
	FeNO	0.750	0.720	0.910
	FVC	0.600	0.001	0.036
	PEF	0.520	0.042	0.150
	$FEV_1\%$	0.340	0.001	0.320
	ACT	0.250	0.001	0.001
	AQLQ	0.090	0.001	0.014
**COPD**	CAT	0.330	0.049	0.038
**Long COVID**	NQ	0.430	0.001	0.049
	dyspnea	0.750	0.032	0.002
	muscular endurance	0.500	0.380	0.049
	Anxiety and sleep-related symptoms	0.910	0.660	0.010
**CO_2-rich centers**	**Parameter**	**Group**	**Time**	**Group x Time**
	$SpCO_2$	0.020	0.040	0.020
	$PetCO_2$	0.200	0.002	0.001
**Total cohort**	**Parameter**	**Group**	**Time**	**Group x Time**
	MIP	0.510	0.020	0.010
	MEP	0.420	0.130	0.049
	NQ scores	0.020	0.001	0.010

For all parameters shown. a non-parametric repeated-measures analysis according to Brunner and Langer was performed. The p-values for the mean group effect (Group). the time course (TIME) and the interaction term Group x Time are shown. Parameters that showed a significant effect were analysed in detail using the Post-hoc Analysis scheme described in Table 3.

Table 3: Scheme of Post-hoc Analysis for Group and Time Effects

Comparison	Level	Test
Within Group Treatment (T)	T1 vs. T2	Wilcoxon signed-rank test
	T1 vs. T3	Wilcoxon signed-rank test
Within Group Control (C)	T1 vs. T2	Wilcoxon signed-rank test
	T1 vs. T3	Wilcoxon signed-rank test
Between Groups T / C	T2 - T1	Mann-Whitney U-Test (MW-U)
	T3 – T1	Mann-Whitney U-Test (MW-U)

T = Treatment. C = Control | T1. T2. T3 = times of measurement. T2 – T1 and T3 – T1 denotes the difference/change for a parameter between the respective two points in time. The MW-U checks for differences in these changes between T and C. Within a group. the Wilcoxon test checks for changes of a parameter between the respective two points in time. To control for Type I error inflation. p-values were adjusted using the Bonferroni-Holm procedure.
